# Adsorption-synergized reduction of Cr(vi) using Gypsum@PANI composite: unraveling mechanism and advanced statistical physics prediction

**DOI:** 10.1039/d6ra04236h

**Published:** 2026-08-04

**Authors:** Samira El Omari, Kamal Ait El Bacha, Abdelaziz Imgharn, Oscar May Tzuc, Youness Abdellaoui, Abdallah Albourine, Karim Benhabib, Mohamed Laabd

**Affiliations:** a Laboratory of Materials and Environment, Faculty of Sciences, Ibnou Zohr University Agadir Morocco m.laabd@uiz.ac.ma; b Eco-Process, Optimization and Decision Support. Jules Verne University of Picardie (EPROAD, UR) France; c Faculty of Engineering, Autonomous University of Campeche Campus V, Av Humberto Lanz, Col. Ex Hacienda Kalá San Francisco de Campeche, Campeche C.P. 24085 Mexico; d Cinvestav Saltillo. Sustainability of Natural Resources and Energy Av. Industria Metalúrgica 1062, Parque Industrial Ramos Arizpe. Ramos Arizpe Coahuila C.P. 25900 Mexico; e CONAHCyT-Cinvestav Saltillo. Sustainability of Natural Resources and Energy Av. Industria Metalúrgica 1062, Parque Industrial Ramos Arizpe. Ramos Arizpe Coahuila C.P. 25900 Mexico; f Laboratory of Industrial Engineering, Energy and Environment (LI3E) SupMTI Rabat Rabat Morocco

## Abstract

Cr(vi) is categorized as a top-priority hazardous contaminant. The subsequent adsorption/reduction of Cr(vi) into Cr(iii) has emerged as a powerful process for detoxifying Cr(vi)-laden waters. Herein, a newly designed Gypsum@Polyaniline (Gypsum@PANI) composite was applied for the detoxification of Cr(vi) ions in aqueous solutions. The as-developed Gypsum@PANI composite exhibited a mesoporous texture abundantly decorated with amine/imine groups. Based on the response surface modeling, the Cr(vi) adsorption effectiveness reached 93.86% under optimized conditions (pH 2, adsorbent dosage of 0.35 g L^−1^, contact time of 4 h, Cr(vi) concentration of 20 mg L^−1^ at 25 °C). Freundlich and pseudo-second order models satisfactorily represented the Cr(vi) adsorption. The maximum uptake capacity was found to be 310.36 mg g^−1^. Advanced statistical physics assessment highlighted that Cr(vi) adsorption proceeded *via* multi-anchorage and multi-molecular pathways. Likewise, the thermodynamic functions proved the spontaneity of the adsorption process, accompanied by energy release and increased disorder. XPS analysis evidenced that the adsorbed Cr(vi) ions were reduced to the less toxic Cr(iii) form. The Gypsum@PANI demonstrated encouraging reusability with a Cr(vi) removal yield of 91% after five successive cycles. Overall, the experimental and theoretical findings asserted that the Gypsum@PANI composite has great potential for the detoxification of Cr(vi) ions in wastewater.

## Abstract 2

Cr(vi) is categorized as a top-priority hazardous contaminant.

## Graphical abstract

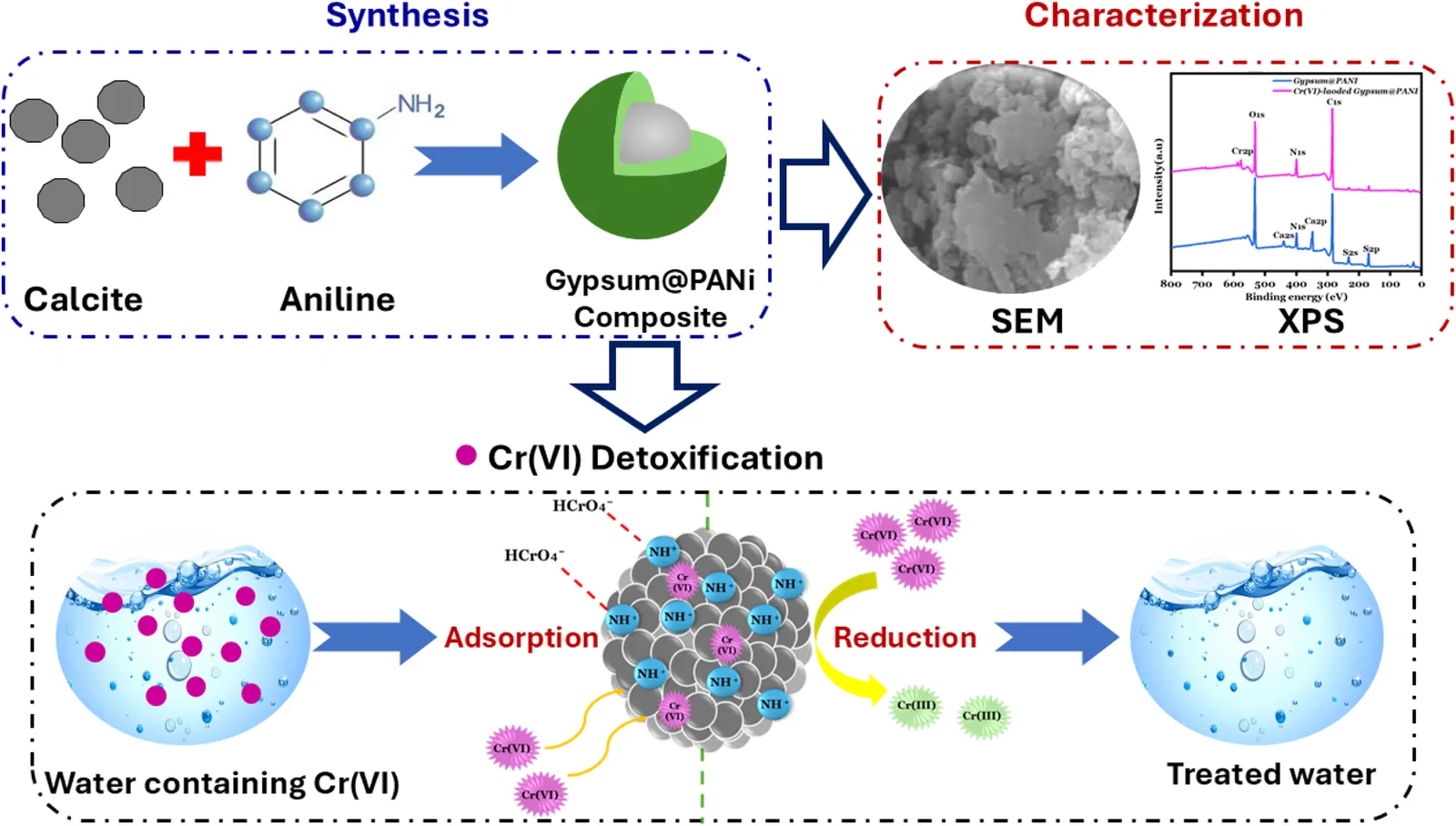

## 1 Introduction

The exponential growth of manufacturing activities and population has caused the release of vast amounts of contaminants into aquatic ecosystems. Among the water contaminants, heavy metals pose severe ecological and health threats, thereby gaining widespread scientific concern.^1^ Hexavalent chromium is regarded as a top-priority contaminant because of its potential toxicity, bioaccumulative nature, and harmful effects (mutagenic and carcinogenic).^2^ Chromium species predominantly originate from several industrial fields (*e.g.*, leather processing, textile, electroplating, stainless steel manufacturing, pigment fabrication, *etc.*).^3^ Chromium occurs mainly in two stable oxidation states, Cr(vi) and Cr(iii), with different toxicological properties.^4^ Cr(iii) cations are almost non-toxic and play beneficial roles in mammalian metabolism. However, Cr(vi) ions are 100-fold more poisonous than Cr(iii) ions. Cr(vi) species can induce tumors, teratogenic effects, DNA damage, and other deleterious impacts.^4^ As reported by the US EPA, the prescribed Cr(vi) concentration in drinking water is restricted to 0.05 mg L^−1^.^5^ Therefore, there is an urgent need for the remediation/detoxification of wastewaters containing high Cr(vi) concentrations to meet regulatory standards.

Currently, various purification technologies have been adopted to remediate Cr(vi)-laden effluents such as electro-reduction,^6^ anion exchange,^7^ photoreduction,^8^ precipitation,^9^ membrane separation,^10^ and adsorption.^11^ Specifically, adsorption is the most employed technique for treating Cr(vi)-contaminated waters owing to its efficacy, minimal cost, flexibility and ease of operation.^11^ The adsorbent is the most crucial component of the adsorption process. Thus, the exploration of effective and sustainable adsorbents is the core objective. In this regard, several adsorbent materials such as carbonaceous materials,^12^ polymeric materials,^13^ metal–organic frameworks,^14^ waste biomass,^15^ zeolites,^16^ and natural clays^17^ have been explored for Cr(vi) removal from water. Since the adsorbed Cr(vi) species can also leach from the sorbent surface, it is crucial to reduce Cr(vi) to Cr(iii). A robust strategy for Cr(vi) remediation involves the reduction of Cr(vi) into Cr(iii), followed by the sequestration of resulting Cr(iii) ions. The N-containing functional moieties can act as potential electron-donor groups for Cr(vi) reduction.^18^ Therefore, the modification of adsorbent surfaces with electron-donating moieties can boost their capability to detoxify Cr(vi)-containing effluents.

Clays are phyllosilicates with layered structures based on tetrahedral silicate layers associated with octahedral aluminum hydroxide layers. They have a high surface area and ion-exchange capacity.^19^ In addition, the high availability and cost-effectiveness of natural clays make their utilization in wastewater treatment economically attractive. Nonetheless, natural clays present low affinity toward anionic contaminants because of their negatively charged lattice framework.^20^ To solve this issue, the surface modification of clay minerals with polycations (*e.g.*, cationic surfactants and cationic polymers) causes a charge reversal, which promotes the adsorption of anionic and neutral contaminants.^21^ For instance, Skorna *et al.* asserted that the Cr(vi) uptake performance of the beidellite clay increased twofold after modification by tetrahexylphosphonium as a cationic surfactant.^20^ In a similar study, El-Kordy *et al.* assessed the Cr(vi) adsorption capacity of raw kaolinitic clay (12 mg g^−1^) and hexadecyltrimethyl ammonium bromide-modified kaolinitic clay (250 mg g^−1^).^22^ In a further investigation, Guo *et al.* employed the polypyrrole (a polymer rich in protonated amine groups) to functionalize bentonite clay for Cr(vi) adsorption from water.^23^ They found that the Cr(vi) uptake increased from 9.5 to 50.9 mg g^−1^ after polypyrrole functionalization.

The conjugated polymers have the advantage of being rich in functional moieties (*e.g.*, amine functions), one of which is polyaniline (PANI).^24^ PANI possesses many protonated amine/imine functions, which can bind contaminants *via* electrostatic forces and H-bonds.^25^ Interestingly, the amino groups of PANI could serve as potential electron donors for reducing Cr(vi) into Cr(iii), strengthening the removal and detoxification performances.^26^ The reusability of PANI (thanks to the reversible doping/de-doping process) is a crucial factor determining its practical feasibility.^27^ Therefore, in the current work, PANI was used to modify the natural clay for Cr(vi) detoxification. Firstly, the Gypsum@PANI core–shell composite was prepared *via* a simple oxidative polymerization route. Secondly, the characteristic features of the Gypsum@PANI composite were assessed using SEM-EDS, XRD, TGA, FTIR, XPS and N_2_ adsorption–desorption. Subsequently, the uptake performance of the Gypsum@PANI composite for Cr(vi) ions was investigated under various operating conditions. Response surface methodology (RSM) was utilized to optimize the Cr(vi) adsorption process. Likewise, the Cr(vi) removal mechanism was clarified through XPS analysis and advanced statistical physics (ASP) formalism. Lastly, the reusability of Gypsum@PANI was evaluated to assess its applicability and economic viability. This work represents the first report on the preparation of a Gypsum@PANI composite for detoxifying highly toxic Cr(vi) ions by a sequential adsorption–reduction process. The Gypsum@PANI composite represents a valuable contribution to the advancement of sustainable functional materials for environmental purposes.

## 2 Experimental and calculation procedures

### 2.1 Gypsum@PANI synthesis

The Gypsum@PANI composite was prepared *via* oxidative polymerization of aniline in the presence of calcite clay particles. The calcite clay was sieved using a 50 µm sieve, rinsed with distilled water and dried at 60 °C. The aniline monomer was previously distilled before use. During the synthesis of Gypsum@PANI, 1 g of the calcite clay was dispersed in 50 mL of HCl (0.5 M). Afterward, 1 mL of aniline was poured into the mixture and vigorously stirred for 1 h. For the oxidizing solution, 22 mmol of Na_2_S_2_O_8_ were dissolved in 50 mL of HCl (0.5 M). Subsequently, Na_2_S_2_O_8_ solution was introduced dropwise into the aniline/calcite clay mixture under stirring for 8 h. The resulting dark-green precipitate was separated, washed with acetone and distilled water, and dried at 60 °C for 3 h.

### 2.2 Characterization

X-ray diffraction (XRD) data of calcite clay and Gypsum@PANI hybrid composite were collected using a Bruker D8 Advance Twin device. XRD analysis was performed over a 2*θ* range of 0–90° using Cu Kα radiation. The morphological properties and elemental constituents of the samples were acquired *via* a scanning electron microscope combined with an energy-dispersive X-ray spectrometer (SEM-EDS) (JEOL, JSM-IT200). Fourier transform infrared (FTIR) spectroscopy was employed to characterize the Gypsum@PANI surface. FTIR analysis was carried out using a Nicolet 5700 spectrometer (Thermo Fisher Scientific) over a 500–4500 cm^−1^ range using a KBr background. The thermogravimetric analysis (TGA) of the pure PANI and Gypsum@PANI composite was accomplished with a thermogravimetric analyzer (NETZSCH STA 449F5 STA449F5B-0167-M). The samples were heated from 20 to 900 °C with a rate of 10°C min^−1^. The X-ray photoelectron spectroscopy (XPS) characterization of Gypsum@PANI pre- and post-Cr(vi) adsorption was done using a Thermo Scientific K-Alpha device. To characterize the Gypsum@PANI surface charge, the potentiometric titration method was applied. Briefly, 30 mg of the Gypsum@PANI composite was mixed with 50 mL of KNO_3_ (0.03 M) solutions with initial pH (pH_i_) values ranging from 2 to 10. After continuous stirring for 48 h, the equilibrium pH_e_ was measured. The point of zero charge (PZC) of the Gypsum@PANI corresponds to the pH_i_ at which ΔpH (pH_e_ − pH_i_) = 0.

### 2.3 Adsorption study and reusability

The Cr(vi) adsorption assays on the Gypsum@PANI composite were conducted in batch mode. Various operational factors such as working pH, Cr(vi) concentration, Gypsum@PANI dosage, adsorption time and temperature were systematically examined. Then, the residual Cr(vi) concentration was assessed at 540 nm by a 2300 UV-vis spectrometer using the 1,5-diphenylcarbazide colorimetric procedure. The Cr(vi) removal yield and the adsorbed amount were determined using the equations presented below:1
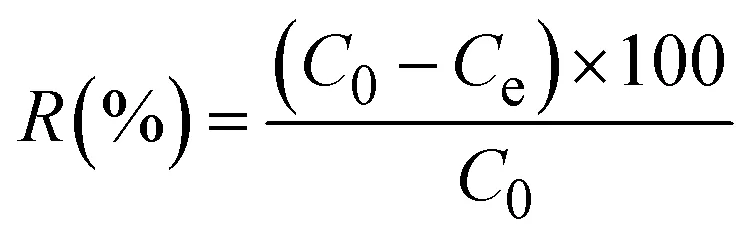
2
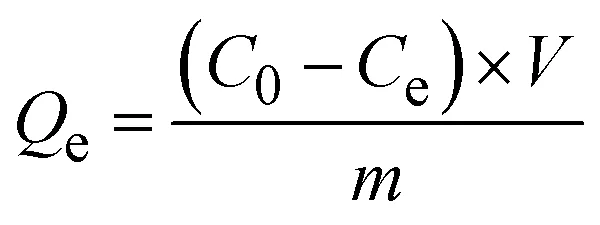


*C*_0_ and *C*_e_ refer to the initial and the residual concentrations of Cr(vi). *V* and *m* refer to the volume of Cr(vi) solution and mass of Gypsum@PANI, respectively.

### 2.4 Modeling the adsorption process by RSM-BBD

RSM-BBD represents a useful approach for designing experiments widely used in process optimization. It is based on statistical experiments to explore the complex interactions between several independent factors and their influence on the process response.^28^ In the current work, RSM-BBD was utilized to optimize the operating conditions to reach the greatest Cr(vi) adsorption performance. In this work, three variables were chosen to evaluate their impact on the Cr(vi) removal yield: Gypsum@PANI dosage (*X*_A_), Cr(vi) concentration (*X*_B_) and solution pH (*X*_C_). A total of 17 experiments were generated using Design-Expert 13 (Table S1). The quadratic polynomial model was used to describe the relationship between the selected factors and the response (eqn (3)).^29^3
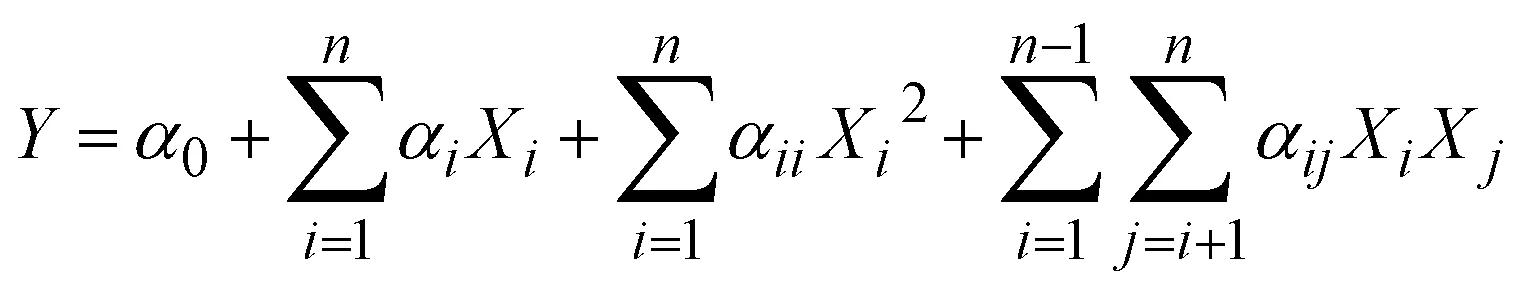
where *Y* is the response (adsorption efficiency). *α*_0_, *α*_*i*_, *α*_*ii*_, and *α*_*ij*_ represent the intercept, linear, quadratic, and interaction terms, respectively. *X*_*i*_ refers to the independent factors (pH, Cr(vi) concentration and Gypsum@PANI dosage).

Analysis of variance (ANOVA) was used to assess the statistical significance of the model terms. A *p*-value below 0.05 signifies that a model term is statistically significant.^30^

### 2.5 ASP analysis

ASP has emerged as an impressive tool to illuminate the binding mechanism at both microscopic and macroscopic levels.^31^ The Cr(vi) adsorption onto Gypsum@PANI was assessed *via* several scenarios using five ASP models.

✓ The first ASP model (ASP1) assumes that the adsorption process occurs as one adsorbed layer, although each surface site can adsorb multiple Cr(vi) ions.

✓ The second ASP model (ASP2) characterizes the formation of one adsorbed layer on the Gypsum@PANI surface containing two distinct receptor sites.

✓ The third ASP model (ASP3) assumes that adsorption proceeds *via* a double-layer coverage of Cr(vi) species onto the Gypsum@PANI surface with identical receptor sites.

✓ The fourth ASP model (ASP4) presumes that adsorption proceeds *via* a double-layer coverage of Cr(vi) species onto the Gypsum@PANI surface with two types of receptor sites.

✓ The fifth ASP model (ASP5) presumes that adsorption occurs *via* a multilayer coverage of Cr(vi) species onto Gypsum@PANI.

The expressions of these ASP models were obtained from the grand ensemble partition function.^32^ The expressions (*Q*_e_ as a function of *C*_e_) of the above-presented ASP models are given below:4
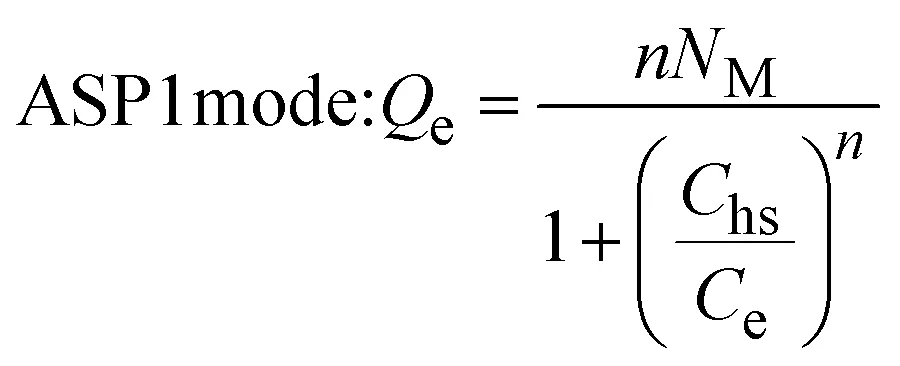
5
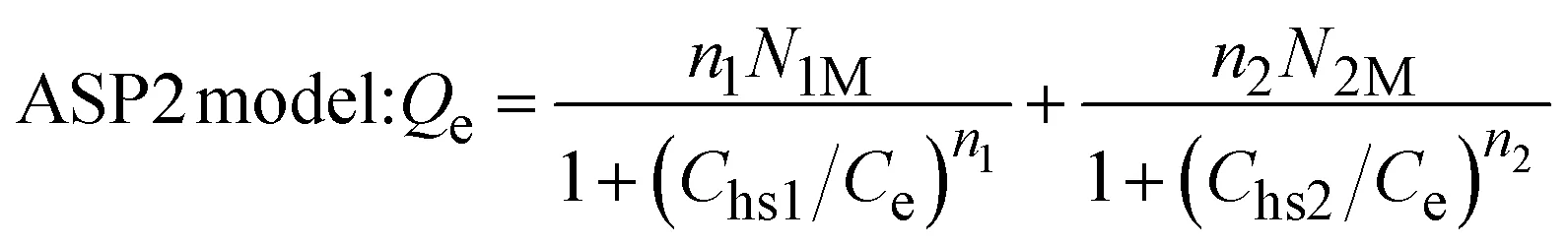
6
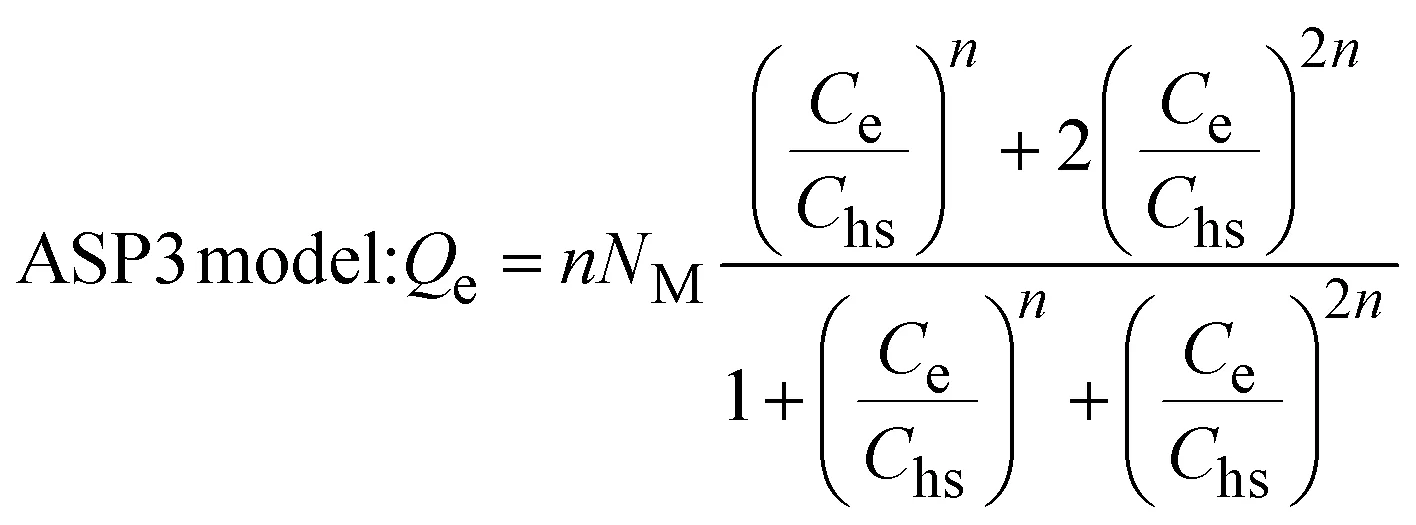
7
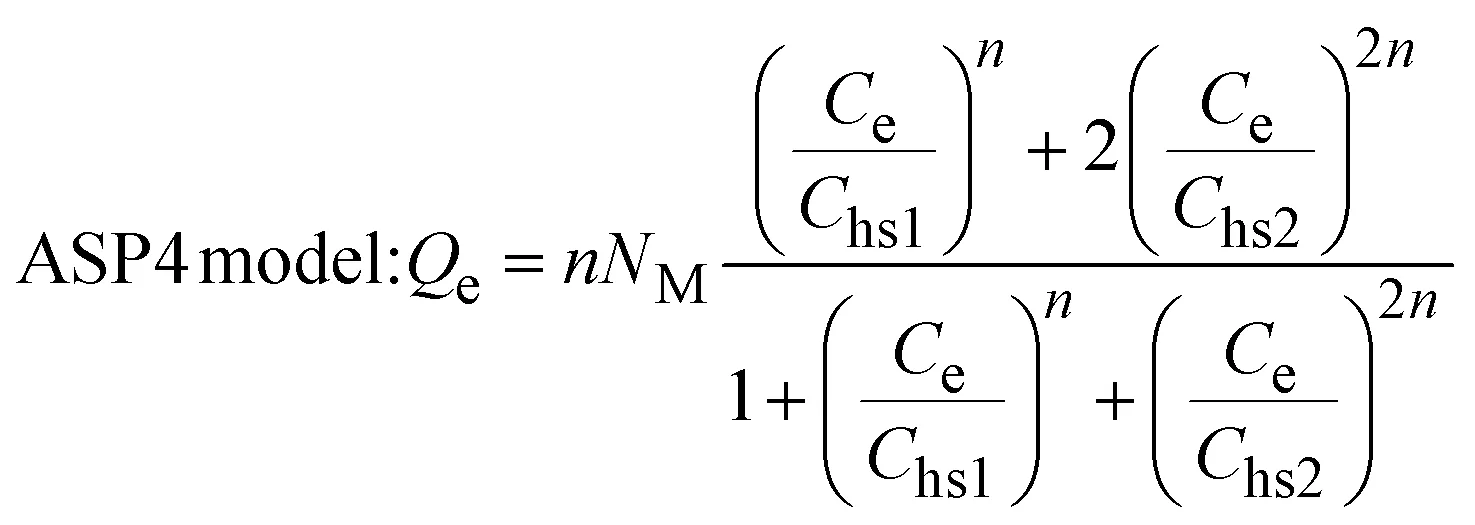
8
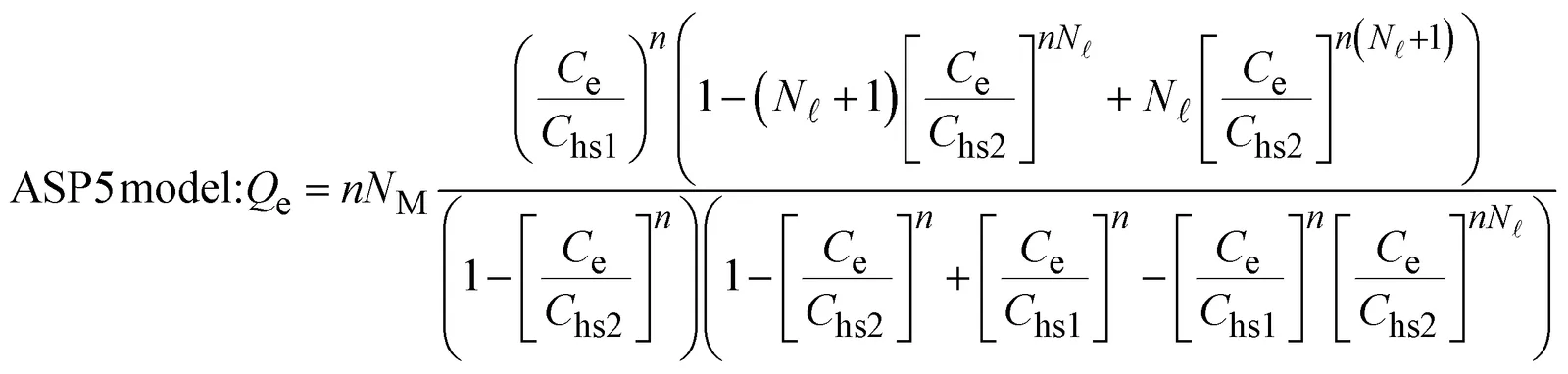


In which *n* denotes the number of Cr(vi) species by each receptor site; *N*_M_ (mg g^−1^) represents the density of receptor sites; *C*_hs_ (mg L^−1^) refers to the half-saturation concentration; 
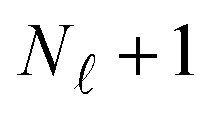
 denotes the number of layers on the Gypsum@PANI surface.

## 3 Results and discussion

### 3.1 Characterization of Gypsum@PANI composite

**Fig. 1:**
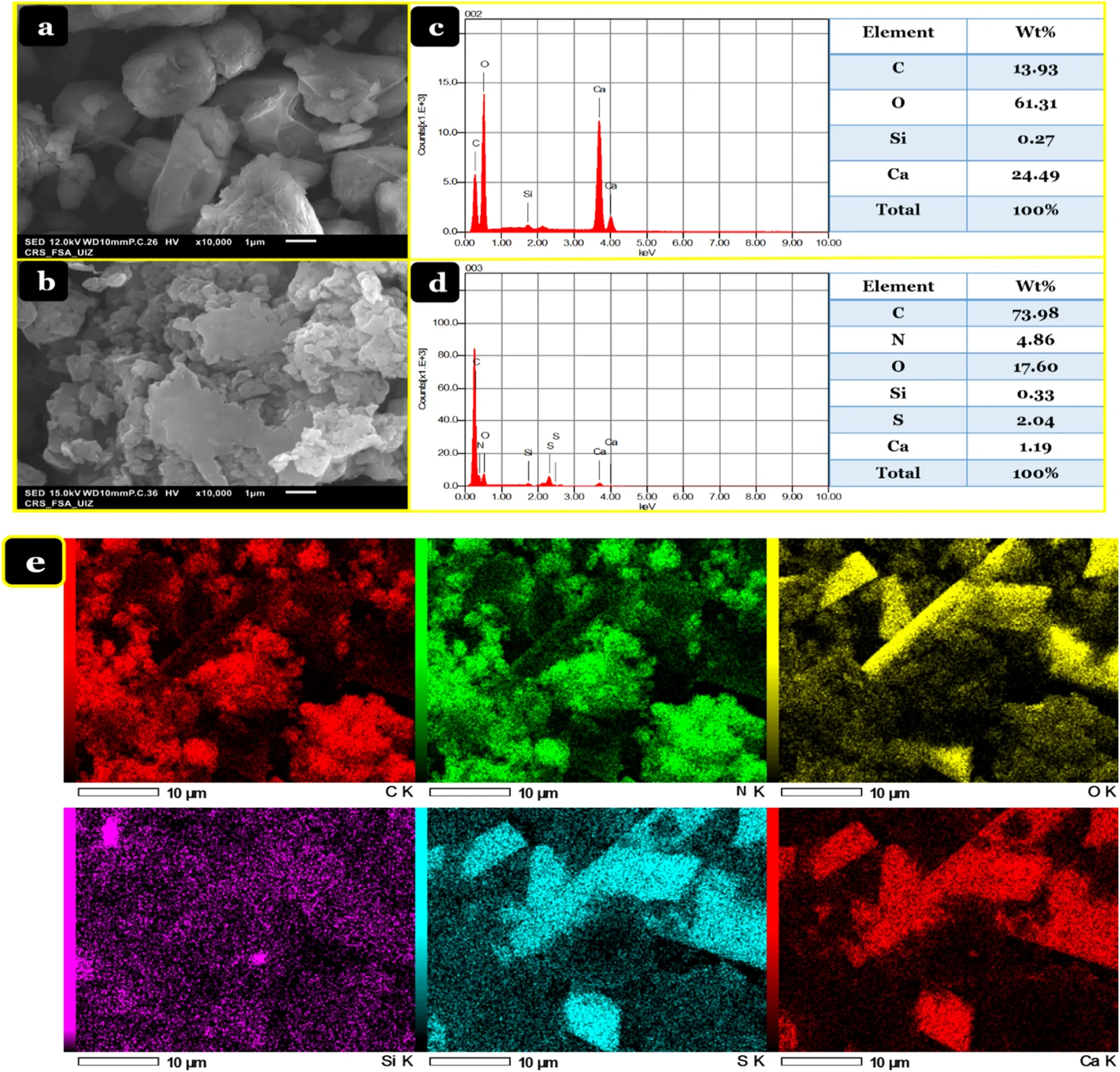
SEM pictures of (a) calcite clay and (b) Gypsum@PANI, EDS spectra of calcite (c) and (d) Gypsum@PANI, and (e) EDS mapping images of Gypsum@PANI.

The morphological features and element composition of the calcite clay and Gypsum@PANI were obtained by SEM-EDS analysis (Fig. 1). The SEM image clearly reveals that calcite exhibits a rhombohedral crystal structure characterized by noticeable agglomeration (Fig. 1(a)).^33^ The Gypsum@PANI micrograph (Fig. 1(b)) displays a significant change in the calcite morphology, with its surface completely coated by a PANI film. Thus, these SEM observations confirm the successful preparation of the Gypsum@PANI composite.

EDS analysis was conducted to complement the SEM observations (Fig. 1(c and d)). The three main peaks detected in Fig. 1(c) correspond to the C, O and Ca elements, which are indicative of the mineral calcite (CaCO_3_). In the EDS spectrum of Gypsum@PANI (Fig. 1(d)), these elements were also detected, but with a lower Ca content and the appearance of a characteristic peak for the S element. Consequently, the calcite phase was converted into gypsum during the synthesis of the composite. In addition to the high C content (73.98%), the characteristic peak of the N element confirms the successful deposition of PANI on the gypsum surface (Fig. 1(d)). The EDS mapping pictures are portrayed in Fig. 1(e). The uniform distribution of C, Ca, O, Si, N and S elements over the Gypsum@PANI surface further confirms the hybridization of PANI with Gypsum.

**Fig. 2:**
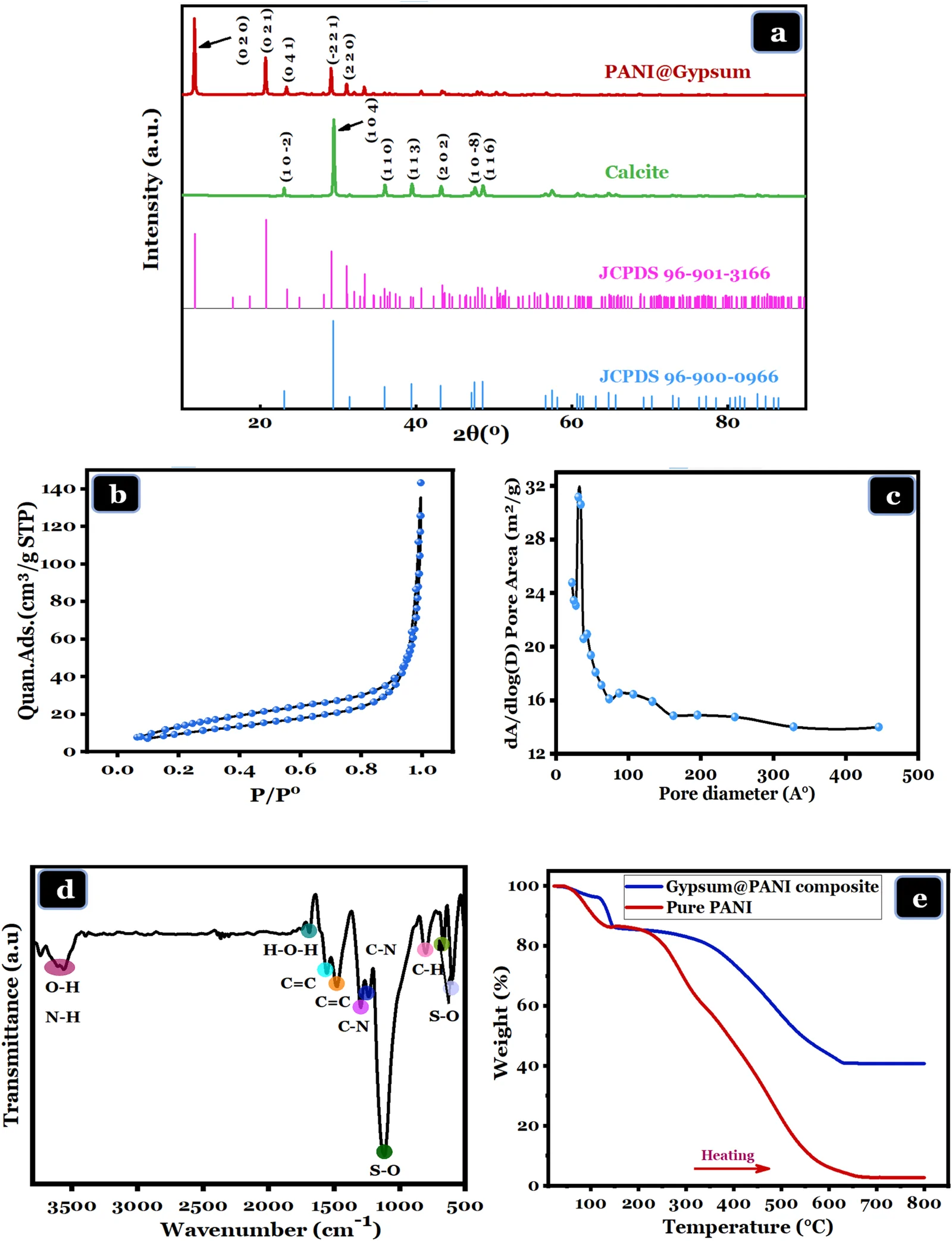
(a) XRD diffractograms of calcite and Gypsum@PANI; (b) isothermal N_2_ adsorption/desorption plots; (c) pore diameter distribution; (d) FTIR spectrum; and (e) TGA curves of pure PANI and Gypsum@PANI.

XRD diffractograms of calcite and Gypsum@PANI are provided in Fig. 2(a). The most prominent peaks corresponding to natural calcite appeared at 2*θ* = 23°, 29.41°, 35.98°, 39.428°, 43.18°, 47.51° and 48.52° with *d*-spacings of 3.85, 3.034, 2.49, 2.28, 2.09, 1.91 and 1.87 Å, which are attributed to the (102̄), (104), (110), (113), (202), (108̄) and (116) crystal planes, respectively. These peaks are characteristic of hexagonal calcite (CaCO_3_) with the space group *R*3̄*c* (JCPDS 96-900-0966). For the Gypsum@PANI sample, the XRD pattern displayed some peaks at 11.65°, 20.75°, 29.15°, 31.12° and 33.38° with *d*-spacings of 7.58, 4.27, 3.061, 2.87 and 2.68 Å, which are characteristic of the (020), (021), (041), (2̄21) and (220) planes, respectively. The good agreement between Bragg's reflections and JCPDS card No. 96-230-0259 confirms the formation of gypsum (CaSO_4_) with a monoclinic crystal structure. The gypsum phase results from the combination of calcium ions and sulfate ions (eqn (12)). The Ca^2+^ cations are produced by dissolution of calcite (CaCO_3_) in HCl (eqn (11)). Besides, the SO_4_^2−^ ions are generated during the oxidation of aniline with Na_2_S_2_O_8_ (eqn (9) and (10)).^34^

9 Na_2_S_2_O_8_ → 2Na^+^ + S_2_O_8_^2−^

10 2S_2_O_8_^2−^ + 2H_2_O → 4SO_4_^2−^ + 4H^+^ + O_2_↑

11 2H^+^ + CaCO_3_ → Ca^2+^ + H_2_0 + CO↑_2_

12 Ca^2+^+ SO_4_^2−^ + 2H_2_O → CaSO_4_·2H_2_O↓

N_2_ sorption–desorption profiles and pore diameter distribution of Gypsum@PANI are given in Fig. 2. According to Fig. 2(b), a small amount of N_2_ is adsorbed under low (*P*/*P*°) conditions with the presence of hysteresis loops. This is characteristic of a type IV adsorption isotherm. Furthermore, the intense peak at *P*/*P*° > 0.8 indicates the capillary condensation within the mesopores, which confirms the mesoporous texture of Gypsum@PANI.^35^ The BET and BJH methods were adopted to characterize the specific surface area, pore volume and pore size. As a result, the Gypsum@PANI composite exhibits a surface area of 61.422 m^2^ g^−1^ and a pore volume of 0.221 cm^3^ g^−1^. In addition, the pore volumes related to the micropores, mesopores, and macropores are 0.0109, 0.0828, and 0.128 cm^3^ g^−1^, respectively. Fig. 2(c) illustrates the pore size distributions of Gypsum@PANI. Therefore, the mesopores and macropores coexist in the Gypsum@PANI structure. The majority of the pores have a pore size of 33 nm, which further proves the mesoporosity of Gypsum@PANI. Hence, Gypsum@PANI exhibits an open mesoporous framework, offering abundant exposed functional sites and boosting the diffusion of Cr(vi) species.

The FTIR spectrum of Gypsum@PANI material is presented in Fig. 2(d). The peaks around 603 and 663 cm^−1^ are attributable to the sulfate groups (SO_4_^2−^) vibrations in gypsum. An additional peak at 1116 cm^−1^ is attributable to asymmetric stretching of the sulfate group (SO_4_^2−^). The band at 1687 cm^−1^ can be correlated to the bending vibrations of H_2_O molecules bound to gypsum.^36^ These findings confirm the transformation of calcite into gypsum during the synthesis of the Gypsum@PANI composite. The coating of PANI on the gypsum surface is further evidenced by the presence of its characteristic absorption bands. The band at 805 cm^−1^ corresponds to the out-of-plane C–H vibrations. The C=C stretching in the benzenoid and quinoid rings of PANI is detected at 1485 and 1556 cm^−1^. The peaks at 1244 and 1298 cm^−1^ result from C–N stretching vibrations. The peak in the range 3400–3600 cm^−1^ arises from an overlap region of O–H (physisorbed water) and N–H of PANI.^36,37^

TGA curves of pure PANI and Gypsum@PANI are shown in Fig. 2(e). The Gypsum@PANI exhibits a three-stage mass loss profile. Initially, the 3.79% mass loss took place between 20 and 115 °C owing to the evaporation of adsorbed water on the Gypsum@PANI composite.^38^ The second mass loss event, occurring between 115 and 150 °C, corresponds to the transformation of gypsum into bassanite (eqn (13)) with a mass loss of 10.08%.^39^ The maximum weight loss observed in the TGA curve is approximately 45.21%, occurring between 150 °C and 632 °C. This weight loss is attributed to the thermal breakdown of PANI chains as well as the loss of crystalline water in bassanite (CaSO_4_·0.5H_2_O).^40,41^ The Gypsum@PANI composite was compared with pure PANI. The weight loss associated with the degradation of the PANI chains began at 214 °C for pure PANI, whereas it started at 285 °C for the Gypsum@PANI composite. These results confirm that the incorporation of gypsum delayed the thermal degradation of PANI chains. This result demonstrated the improved thermal stability of the Gypsum@PANI composite. The enhanced thermal stability may be attributed to the interactions between PANI and Gypsum, restricting the thermal motion of the PANI chains in the Gypsum@PANI composite.^42^

13 CaSO_4_·2H_2_O + Heating → CaSO_4_·0.5H_2_O(bassanite) + 1.5H_2_O

### 3.2 Cr(vi) removal study

#### 3.2.1 Influence of Gypsum@PANI dosage and pH

**Fig. 3:**
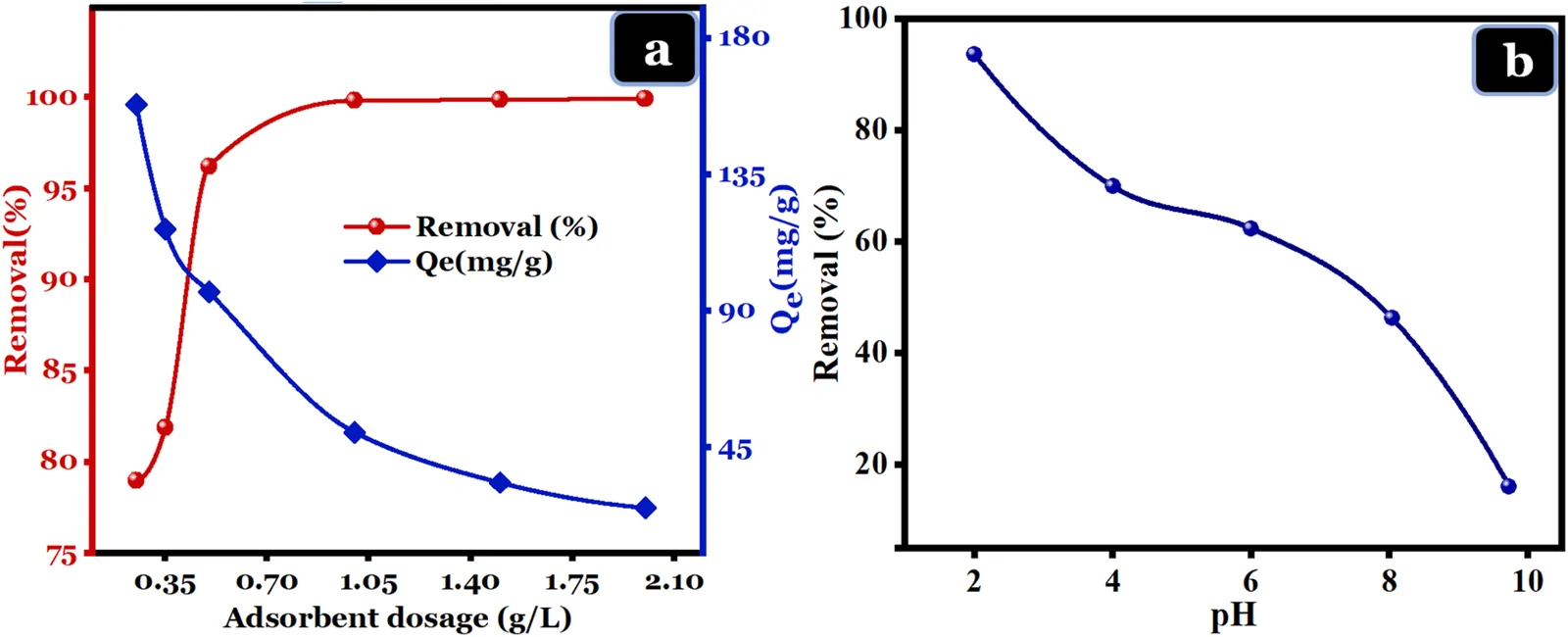
Influence of (a) Gypsum@PANI dosage (pH 2; adsorption time = 4 h; [Cr] = 20 mg L^−1^; 25 °C) and (b) solution pH (Gypsum@PANI dosage = 0.35 g L^−1^; adsorption time = 4 h; [Cr] = 20 mg L^−1^; 25 °C) on Cr(vi) adsorption.

In this section, the focus is on determining the role of the Gypsum@PANI dosage on Cr(vi) adsorption performance. As portrayed in Fig. 3(a), the Cr(vi) removal performance of the Gypsum@PANI composite is significantly improved by increasing the Gypsum@PANI dosage from 0.15 to 0.35 g L^−1^. This suggests that increasing the Gypsum@PANI dosage provided more binding sites for Cr(vi) adsorption. Beyond 0.35 g L^−1^, no considerable influence on Cr(vi) adsorption yield was observed. This trend indicates that most of the Cr(vi) ions had already been adsorbed. In contrast, the adsorption capacity gradually decreased with increasing Gypsum@PANI dosage. This trend can be attributed to the relative abundance of unoccupied active sites resulting from a high Gypsum@PANI : Cr(vi) ratio. Moreover, at higher Gypsum@PANI dosages, the agglomeration of the Gypsum@PANI particles may reduce the accessibility of active sites.^43^ The optimal Gypsum@PANI dosage was 0.35 g L^−1^.

The adsorption process is sensitive to pH, as it is directly related to the ionization states of both the sorbent and the pollutant. The influence of pH on Cr(vi) adsorption was assessed in the range between 2 and 10. According to Fig. 3(b), the highest Cr(vi) adsorption yield of 93.61% was reached at pH 2 and then dropped sharply to 16.13% at pH 10. In other words, the Gypsum@PANI material demonstrates superior effectiveness for Cr(vi) removal at low pH. To explain this finding, the PZC of Gypsum@PANI was found to be 2 (Fig. S1). This value suggests that the Gypsum@PANI exhibits a positive charge at pH < PZC, while at pH > PZC the surface charge is negative. Under acidic conditions, H^+^ ions protonate the amino functions of Gypsum@PANI. This positive surface charge promotes Cr(vi) adsorption by electrostatic interactions (eqn (14) and (15)).^44^ At alkaline pH, OH^−^ ions compete with Cr(vi) species for the active sites, thereby inhibiting Cr(vi) adsorption.^44,45^

14 –NH_2_ + H^+^ → –NH_3_^+^

15 –NH_3_^+^ + HCrO_4_^−^ → –NH_3_^+^⋯HCrO_4_^−^

#### 3.2.2 Kinetic and isothermal modeling

**Fig. 4:**
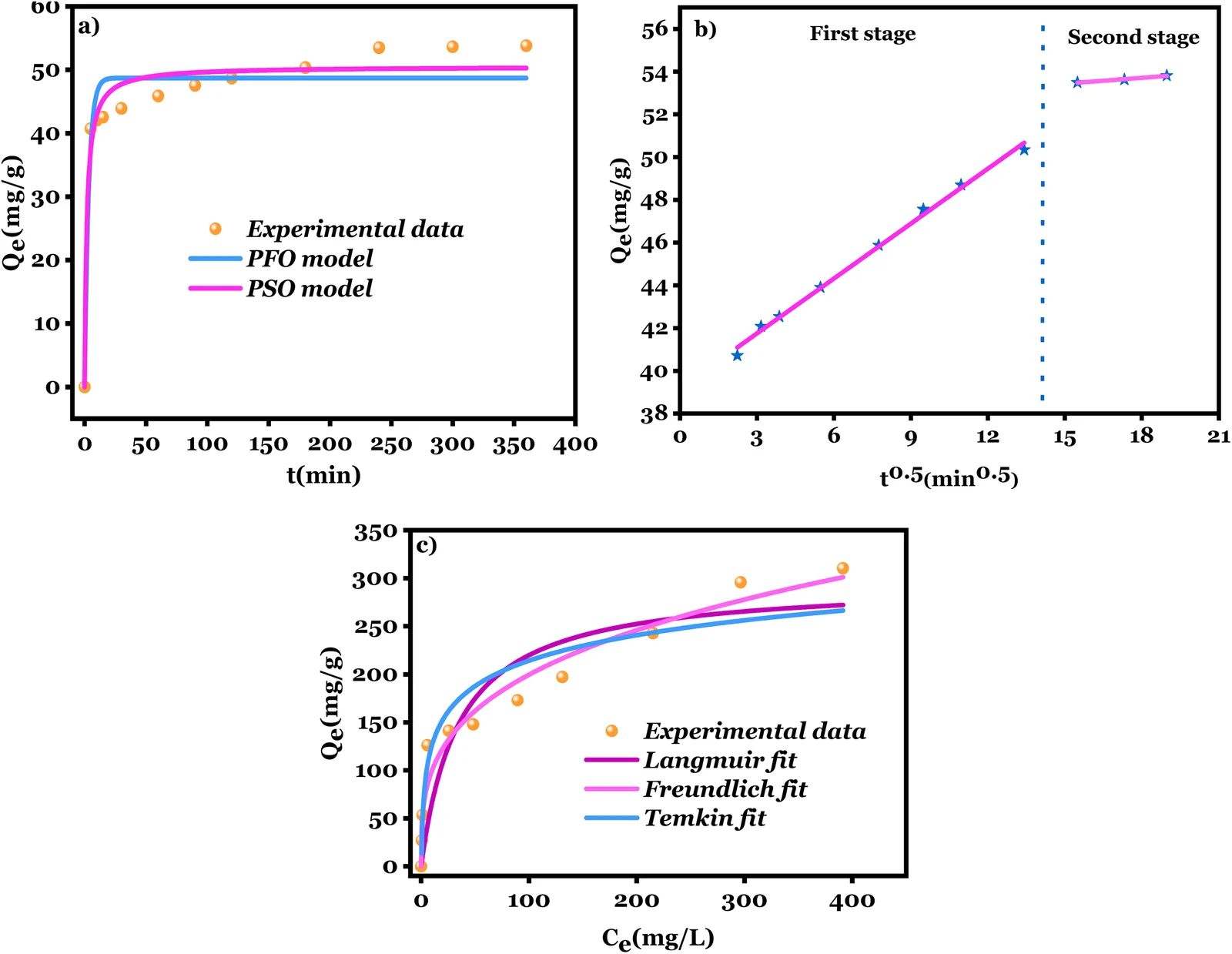
(a) PFO and PSO nonlinear plots, (b) IPD plots and (c) isotherm fitting curves for Cr(vi) adsorption on Gypsum@PANI.

To assess the adsorption kinetics, the adsorption experiments were accomplished over a time range of 5–360 min. From Fig. 4(a), it is evident that the Cr(vi) adsorption rate increased substantially during the first 5 min, followed by a slowdown to achieve an equilibrium state after 240 min. This behavior might be due to the occurrence of ample free receptor sites on the Gypsum@PANI composite at the beginning of the adsorption process. With prolonged adsorption time, the number of free receptor sites gradually decreased to reach the equilibrium state.^46^

In this study, the pseudo-first order (PFO), pseudo-second order (PSO) and intraparticle diffusion (IPD) equations were employed to appraise actual findings (Fig. 4(a)). The nonlinear equations, correlation coefficients (*R*^2^) and kinetic constants are presented in Table 1. Accordingly, the highest *R*^2^ value corresponds to the PSO model (*R*^2^ = 0.959), suggesting that it most accurately describes the Cr(vi) adsorption onto Gypsum@PANI. The good agreement between the theoretical (*Q*_e.PSO_ = 50.533 mg g^−1^) and experimental (*Q*_e.Exp_ = 53.81 mg g^−1^) adsorption capacities further validates the reliability of the PSO model.

Kinetic and isothermal constants for Cr(vi) adsorption onto Gypsum@PANI

**Table:** 

Kinetic models						
*Q*_e.Exp_	PFO equation			PSO equation		
	*Q*_*t*_ = *Q*_e.PFO_(1 − exp(−*k*_PFO_*t*))			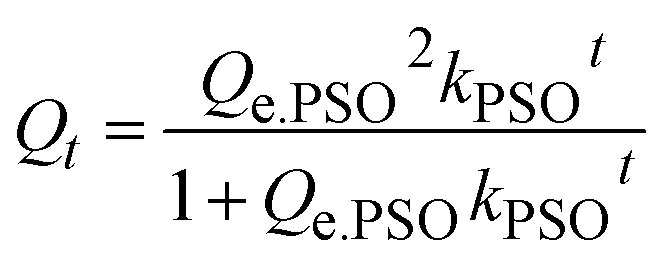		
	*k*_PFO_	*Q*_e.PFO_	*R* ^2^	*k*_PSO_	*Q*_e.PSO_	*R* ^2^
53.81	0.302	48.719	0.921	0.0112	50.533	0.959

**Table:** 

IPD equation: *Q*_*t*_ = *k*_IPD_*t*^1/2^ + *β*					
First stage			Second stage		
*k*_IPD1_	*β*_1_	*R* ^2^	*k*_IPD2_	*β*_2_	*R* ^2^
0.856	39.179	0.994	0.0914	52.064	0.975

**Table:** 

Isotherm models									
*Q*_m.Exp_ (mg g^−1^)	Langmuir 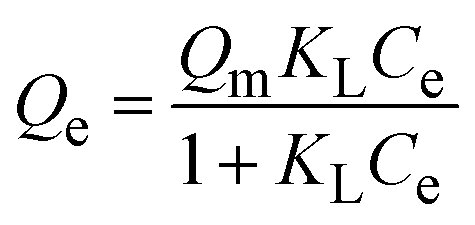			Freundlich *Q*_e_ = *K*_F_*C*_e_^1/^*n*^_F_^			Temkin *Q*_e_ = *B* ln(*AC*_e_)		
	*Q*_m_	*K*_L_	*R* ^2^	*n*_F_	*K*_F_	*R* ^2^	*A*	*B*	*R* ^2^
310.36	296.23	0.028	0.834	3.318	49.811	0.963	2.707	38.242	0.913

The mass transport process during the Cr(vi) adsorption was assessed using the IPD model. Fig. 4(b) shows that the IPD model exhibits a two-segment profile, implying that the Cr(vi) adsorption is a two-step process. The first step represents the migration of Cr(vi) ions from the solution to the external surface of Gypsum@PANI. The second step involves the penetration of Cr(vi) species inside the Gypsum@PANI pores. As shown in Table 1, a pronounced decrease in the Cr(vi) adsorption rate (*k*_IPD_) occurred when going from the first to the second step. However, the boundary layer thickness (*β*) increased over time. This behavior suggests that film diffusion is of prime importance in the Cr(vi) mass transport pathway.^47^

The equilibrium results were analyzed by Langmuir, Freundlich and Temkin isothermal models (Fig. 4(c)). As a result, the Freundlich isotherm (*R*^2^ = 0.963) appears to be more accurate for predicting the Cr(vi) adsorption. Therefore, Cr(vi) adsorption follows a multilayer coverage onto Gypsum@PANI.^48^ It is noteworthy that 0 < 1/*n*_F_ < 1, which indicates that the Cr(vi) adsorption is favorable.^49^ The performance of Gypsum@PANI for Cr(vi) removal was compared with that of other materials studied by other authors. Table 2 summarizes the uptake capacities (*Q*_m_) obtained for each adsorbent. Accordingly, the Gypsum@PANI composite demonstrates competitive performance for Cr(vi) removal compared with the other reported adsorbents. Thus, it is evident that Gypsum@PANI represents a potential option for Cr(vi) removal.

**Table 2:** Cr(vi) uptake capacities of Gypsum@PANI and other adsorbents

Material	*Q*_max_ (mg g^−1^)	Reference
PANI@MIBWS	162.02	26
CeFe@C	75.11	48
PANI@WH	31.45	50
Iron oxide-modified biochar composite	24.37	51
Mg_2_Al-LDH@WT composite	28.82	52
KHCO_3_-activated biochar	153.37	53
Fluorine-free niobium carbide	93.6	54
AOPAN/PANI-16 NFM	299.56	55
UiO-66-AMmpz	291.2	56
Gypsum@PANI	310.36	This work

#### 3.2.3 RSM-BBD modeling

In the present study, the BBD combined with RSM was adopted to analyze individual and mutual effects of the three operating factors: Gypsum@PANI dosage, pH and Cr(vi) concentration. Table S1 presents the BBD experimental design runs and the actual Cr(vi) removal efficiency. The Cr(vi) adsorption was modeled employing the following RSM-BBD polynomial formula:

16 *Y*(%) = +19.88 + 15.07 × *X*_A_ − 12.52 × *X*_B_ − 24.83 × *X*_c_ − 10.35 × *X*_AB_ − 16.15 × *X*_AC_ − 2.20 × *X*_BC_ − 3.78 × *X*_A_^2^ + 12.23 × *X*_B_^2^ + 24.16 × *X*_C_^2^

ANOVA was performed to assess the appropriateness of the RSM-BBD model to predict the actual findings. ANOVA is reported in Table 3. As a result, the high *F*-value (45.96) and very low *p*-value (<0.0001) prove that the developed RSM-BBD model is significant. Since the lack of fit was insignificant (*F*-value = 2.98), the statistical model is suitable for providing accurate insights into the Cr(vi) adsorption process.^50^ Except for the *X*_B/C_ and *X*_A_^2^ terms, the *p*-values of all other model terms (*X*_A_, *X*_B_, *X*_C_, *X*_A/B,_*X*_A/C,_*X*_B_^2^ and *X*_C_^2^) were less than 0.05. This signifies that these model terms exhibited a major effect on Cr(vi) adsorption onto Gypsum@PANI. In addition, the *X*_C_-factor (Cr(vi) concentration) was the most important operational parameter because it has the highest *F*-value. So, the importance of the operational factors on the Cr(vi) removal effectiveness is as follows:Cr(vi) concentration > Gypsum@PANI dosage > pH

**Table 3:** ANOVA for the RSM-BBD model

Source		Sum of squares	d*f*	Mean square	*F*-value	*p*-value
Model		12733.65	9	1414.85	45.96	<0.0001
*X*_A_-Gypsum@PANI dosage		1816.77	1	1816.77	59.02	0.0001
*X*_B_-pH		1253.62	1	1253.62	40.72	0.0004
*X*_C_-Cr(vi) concentration		4932.34	1	4932.34	160.23	<0.0001
*X*_A_ *X*_B_		428.11	1	428.11	13.91	0.0074
*X*_A_ *X*_C_		1043.83	1	1043.83	33.91	0.0006
*X*_B_ *X*_C_		19.30	1	19.30	0.63	0.4545
*X*_A_^2^		59.77	1	59.77	1.94	0.2061
*X*_B_^2^		629.43	1	629.43	20.45	0.0027
*X*_C_^2^		2457.50	1	2457.50	79.83	<0.0001
Residual		215.49	7	30.78		
Lack of fit		148.84	3	49.61	2.98	0.1596
Pure error		66.64	4	16.66		
Cor total		12949.14	16			
Statistics	*R* ^2^	Adj-*R*^2^		Pred-*R*^2^	Adeq. precision	
	0.984	0.962		0.808	20.029	

To further validate the developed statistical model, the predicted% removal was plotted *versus* the actual% removal. From Fig. S2(a), it is obvious that the predicted values for Cr(vi) removal correlate well with the observed data. Similarly, Fig. S2(b) reveals that the residuals were normally distributed within the expected range. Furthermore, the correlation coefficient (*R*^2^) was found to be 0.983. The *R*^2^ value was nearly equal to 1, which reflects the model's reliability. In addition, the adequate precision has a high value of 20.029 (>4), implying an adequate signal-to-noise.^37^

**Fig. 5:**
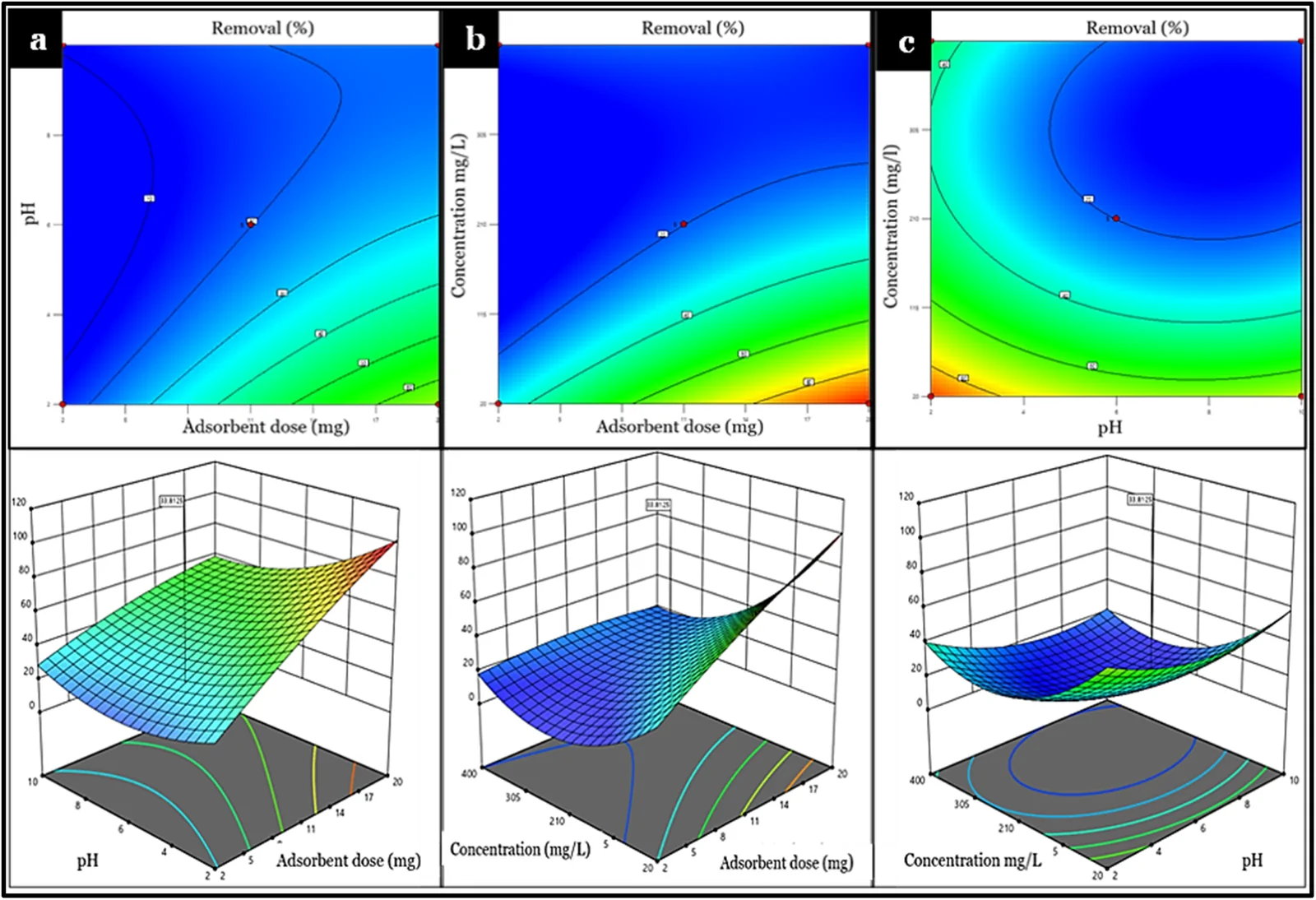
3D surface and 2D contour plots for Cr(vi) removal with respect to (a) Gypsum@PANI dosage and pH, (b) Gypsum@PANI dosage and Cr(vi) concentration and (c) pH and Cr(vi) concentration.

An in-depth study of the interactive effects of input parameters on the Cr(vi) adsorption efficiency requires the use of 3D and 2D plots (Fig. 5). Fig. 5(a) displays the mutual effect of pH and Gypsum@PANI dosage. Cr(vi) was removed up to 96% at pH 2 and progressively decreased to reach 12.14% at pH 10. This behavior may result from the progressive decrease in the positively charged sites on the Gypsum@PANI surface. Consequently, the electrostatic attraction weakens, which decreases the Cr(vi) removal yield.^35,57^ The Gypsum@PANI dosage is another key operating factor. Increasing the Gypsum@PANI mass from 2 to 20 mg raises the number of accessible receptor sites, thus enhancing Cr(vi) adsorption yield from 17.41% to 95.44%.^30^Fig. 5(b) represents the mutual impact of Cr(vi) concentration and Gypsum@PANI dosage. The 3D and 2D contour plots clearly show that raising Cr(vi) concentration negatively affects the Cr(vi) removal yield. This trend is due to the progressive filling of binding sites. Overall, the highest Cr(vi) adsorption yield of 93.86% was attained under the optimized conditions: 20 mg of Gypsum@PANI, pH 2, 20 mg L^−1^ of Cr(vi) for 4 h at 25 °C.

#### 3.2.4 Physicochemical interpretation

The Cr(vi) adsorption was assessed at three operating temperatures (298, 308 and 318 K) using five ASP models. The *R*^2^ and RMSE were used to choose the ASP model that best describes Cr(vi) adsorption onto Gypsum@PANI. As shown in Table 4, the ASP2 model demonstrated the highest consistency with the actual results, as upheld by its superior *R*^2^ and lowest RMSE values. Thus, Cr(vi) adsorption proceeded *via* a monolayer coverage on the Gypsum@PANI surface with two binding sites. Based on the ASP2 model, the Cr(vi) binding mechanism onto Gypsum@PANI was examined using physicochemical parameters and configurational thermodynamic functions.

ASP parameters for Cr(vi) adsorption onto Gypsum@PANI

**Table:** 

298 K	*n*_1_	*Q*_1_	*C*_hs1_	*N*_1_	d*E*_1_	*n*_2_	*Q*_2_	*C*_hs2_	*N*_2_	d*E*_2_	*R* ^2^	RMSE
ASP1	0.547	336.578	36.736	615.326	14.252	—	—	—	—	—	0.9046	30.270
ASP2	0.337	328.686	96.052	975.464	16.634	1.287	192.825	399.798	149.782	20.169	0.9731	16.061
ASP3	0.444	334.357	386.718	752.542	20.087	—	—	—	—	—	0.9191	27.863
ASP4	0.440	305.710	105.506	694.380	16.867	—	—	395.748	—	20.144	0.9414	23.723
ASP5	0.400	128.103	112.434	320.257	17.024	—	—	—	—	—	0.9110	29.230

**Table:** 

308 K	*n*_1_	*Q*_1_	*C*_hs1_	*N*_1_	d*E*_1_	*n*_2_	*Q*_2_	*C*_hs2_	*N*_2_	d*E*_2_	*R* ^2^	RMSE
ASP1	0.567	524.948	132.559	926.380	18.017	—	—	—	—	—	0.8874	41.382
ASP2	0.502	366.634	109.646	730.334	17.531	1.500	192.089	275.711	128.060	19.893	0.9290	32.944
ASP3	0.485	375.927	364.996	775.107	20.612	—	—	—	—	—	0.9133	36.306
ASP4	0.457	380.718	332.565	832.679	20.374	—	—	374.937	—	20.681	0.9159	35.777
ASP5	0.500	175.162	336.359	349.987	20.403	—	—	—	—	—	0.8530	47.291

**Table:** 

318 K	*n*_1_	*Q*_1_	*C*_hs1_	*N*_1_	d*E*_1_	*n*_2_	*Q*_2_	*C*_hs2_	*N*_2_	di_2_	*R* ^2^	RMSE
ASP1	0.596	595.194	128.483	999.197	18.519	—	—	—	—	—	0.8925	45.977
ASP2	0.350	422.076	129.404	1206.383	18.538	1.453	186.096	151.475	128.063	18.955	0.9429	33.514
ASP3	0.540	405.012	327.746	749.634	20.996	—	—	—	—	—	0.9146	40.988
ASP4	0.506	399.364	173.006	789.223	19.306	—	—	334.006	—	21.046	0.9193	39.841
ASP5	0.402	272.570	291.396	677.478	20.685	—	—	—	—	—	0.8919	46.099

#### 3.2.5 Statistical physics parameters

**Fig. 6:**
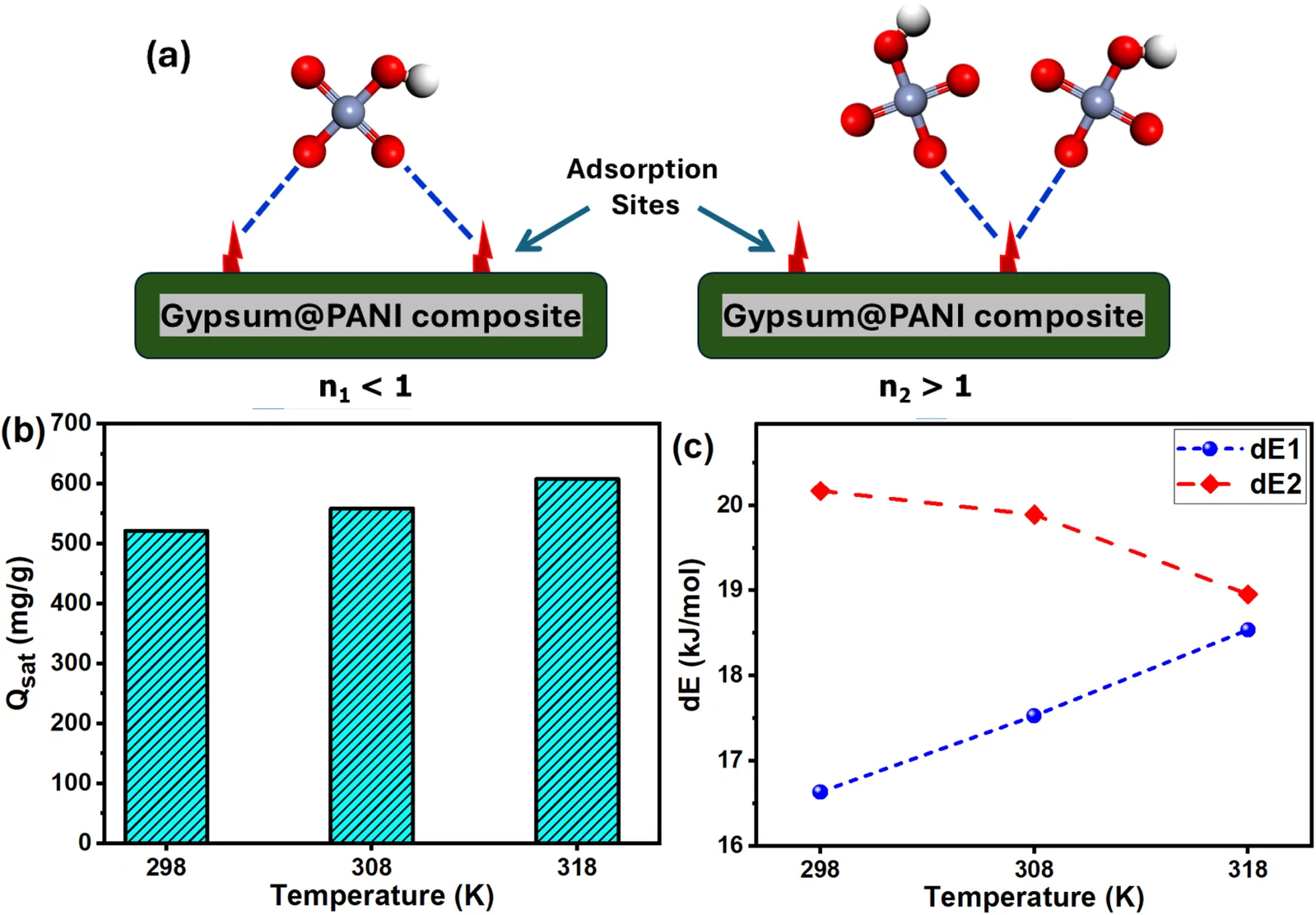
(a) Schematic illustration of the anchoring of Cr(vi) species on the Gypsum@PANI and variation of (b) adsorbed amount at saturation and (c) adsorption energy at various temperatures.

The stereographic analysis is critical for understanding the Cr(vi) attachment tendencies on the Gypsum@PANI surface. Table 4 summarizes the values of stoichiometric coefficients (*n*_1_ and *n*_2_) at different temperatures. In fact, *n* > 1 indicates the presence of multiple docked Cr(vi) ions per receptor site. Conversely, *n* < 1 reveals a multi-anchorage adsorption of each Cr(vi) ion across multiple binding sites.^58^ It is evident from Table 4 that the *n*_1_ values are inferior to unity, suggesting that multiple binding sites can share each Cr(vi) ion. This mechanism is called multianchorage adsorption. In contrast, the values of *n*_2_ are greater than unity, demonstrating that multiple Cr(vi) species are bound to each binding site. This phenomenon is known as multimolecular docking. Both multianchorage and multimolecular mechanisms are schematized in Fig. 6(a).

Fig. 6(b) depicts the effect of temperature on the total saturation adsorption quantity (*Q*_sat_ = *Q*_1_ + *Q*_2_ = *n*_1_ × *N*_1_ + *n*_2_ × *N*_2_). As a result, the increment in Cr(vi) uptake capacity with temperature demonstrates that the adsorption process is endothermic. In addition, the increment in temperature increases the Cr(vi) mobility and promotes swelling within the inner structure of the Gypsum@PANI composite.^58^ As a consequence, increasing temperature boosts the penetration of Cr(vi) oxyanions within the Gypsum@PANI pores.

Fig. 6(c) depicts the evolution of adsorption energies (d*E*_1_ and d*E*_2_) with temperature. Accordingly, the adsorption energies were less than 40 kJ mol^−1^, implying that Cr(vi) adsorption is controlled by weak intermolecular interactions (low energy barriers).^59^ Thus, increasing temperature boosts the ability of Cr(vi) ions to surmount energy barriers. These findings suggest the potential regeneration of Cr(vi)-loaded Gypsum@PANI.^60^ The statistical physics parameters reinforce the heterogeneous nature of Cr(vi) adsorption (multianchorage and multimolecular), corroborating the Freundlich model. Besides, the low adsorption energies suggest that the Cr(vi) adsorption is physisorption. This suggestion is consistent with pH-dependent adsorption, which demonstrates that electrostatic forces play a pivotal role in the Cr(vi) binding mechanism.

#### 3.2.6 Thermodynamic functions

The Gibbs free energy sheds light on the feasibility of the adsorption reaction. According to the ASP2 model, this fundamental thermodynamic function is expressed as:^61^17
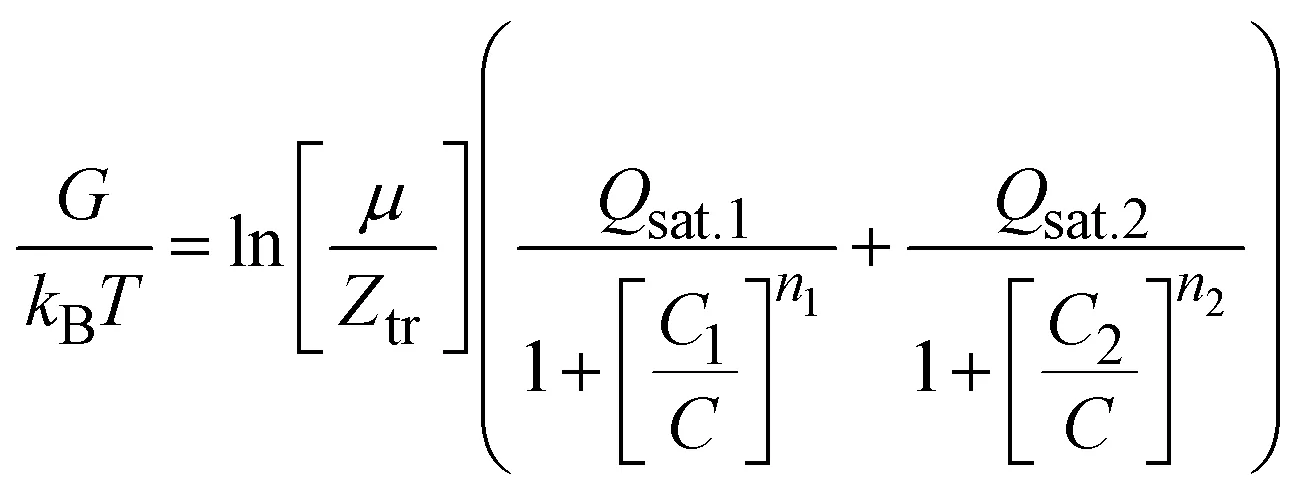


**Fig. 7:**
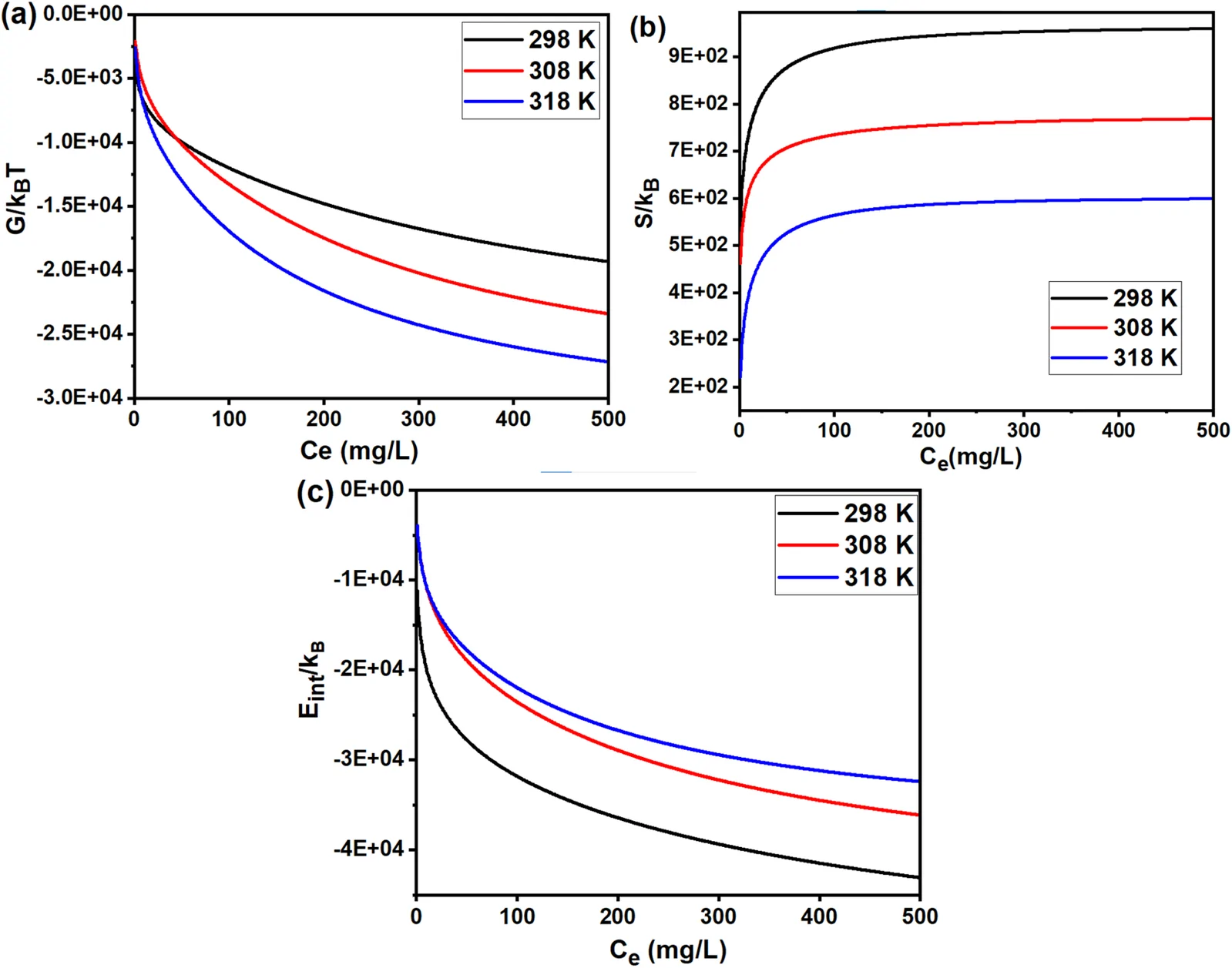
(a) 
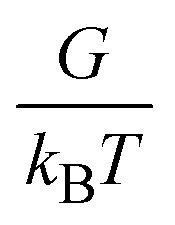
, (b) 
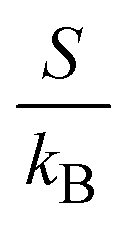
 and (c) 
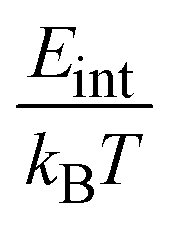
 thermodynamic functions for Cr(vi) adsorption.

Fig. 7(a) illustrates the evolution of 
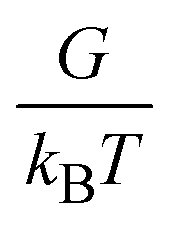
*vs.* Cr(vi) concentration. It is worthwhile noting that the values of 
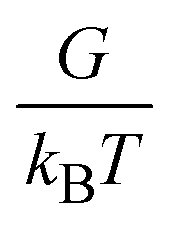
 function are negative over the studied temperature range. This outcome confirms that the Cr(vi) adsorption proceeds spontaneously. Besides, at relatively high equilibrium Cr(vi) concentrations, the 
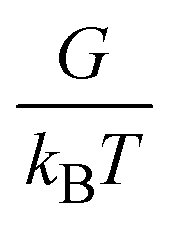
 function reaches a plateau. This trend indicates that further addition of Cr(vi) ions does not substantially influence the overall energy of the adsorption process.^62^ In addition, a rise in temperature results in more negative 
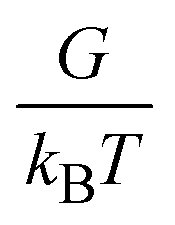
 values, demonstrating that higher temperatures favor Cr(vi) adsorption.

The entropy 
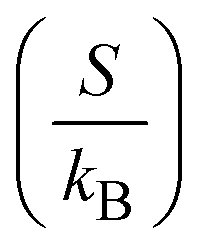
 function sheds light on the order-disorder in the solution-adsorbent system. The 
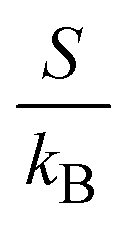
 function for the ASP2 model is formulated as follows:^61^18
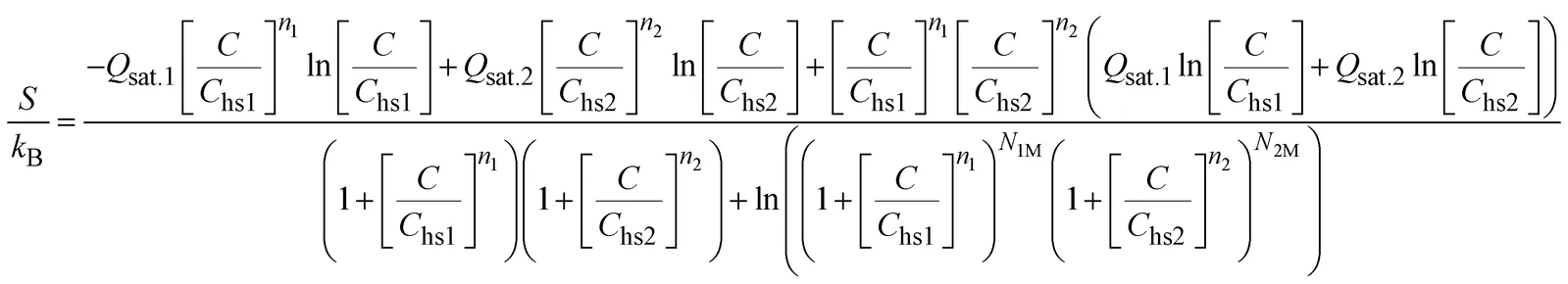


Fig. 7(b) shows the evolution of 
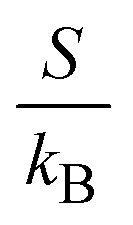
 function *versus* Cr(vi) concentration. When the Cr(vi) concentration increases, the 
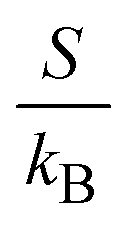
 function increases rapidly until it attains a plateau at higher concentrations. The increment in configurational entropy (increased randomness) may be caused by increased adsorbent–adsorbate collisions.^63^

The internal energy 
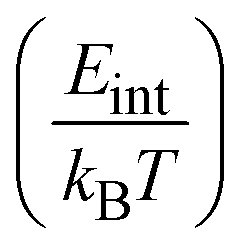
 function corresponds to the total energy of the adsorption process. According to the ASP2 model, the internal energy function is expressed as:^64^19
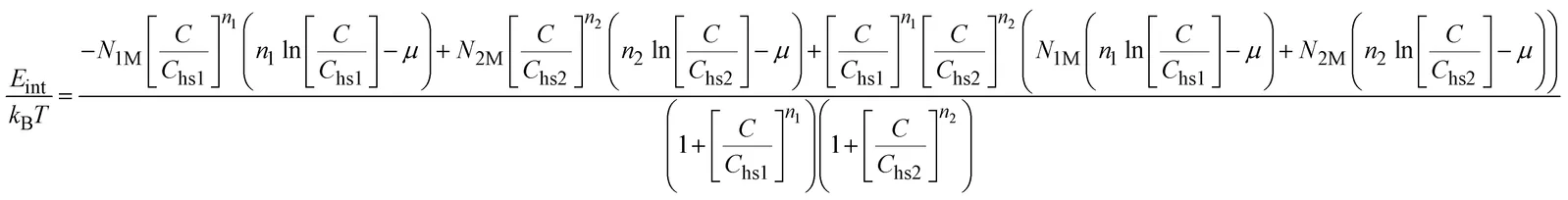


The internal energy function is shown in Fig. 7(c). As a result, the negative values of 
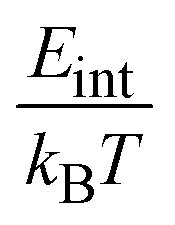
 indicate that the adsorption process releases energy to the outside. This finding suggests a strong affinity of Gypsum@PANI with Cr(vi) oxyanions.^65^ It is also noteworthy that the internal energy values decline with increasing Cr(vi) equilibrium concentration. At low Cr(vi) concentrations, the Cr(vi) species preferentially bind to the highly active sites, causing a pronounced drop in 
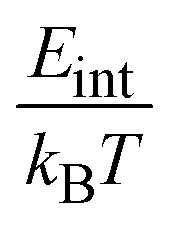
 values. As Cr(vi) concentration increases, the Cr(vi) species adsorb on the weaker receptor sites, resulting in a minor decrease in 
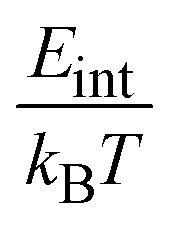
 values.^66^ Interestingly, the 
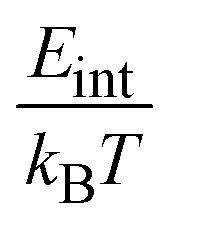
 function increased with operating temperature. This behavior is inferred from the decrease in thermal collisions.^67^ Overall, the thermodynamic functions confirm that Cr(vi) adsorption is a favorable process with strong adsorbate–adsorbent affinity. This conclusion is consistent with adsorption isotherm analysis.

#### 3.2.7 Cr(vi) detoxification mechanism

**Fig. 8:**
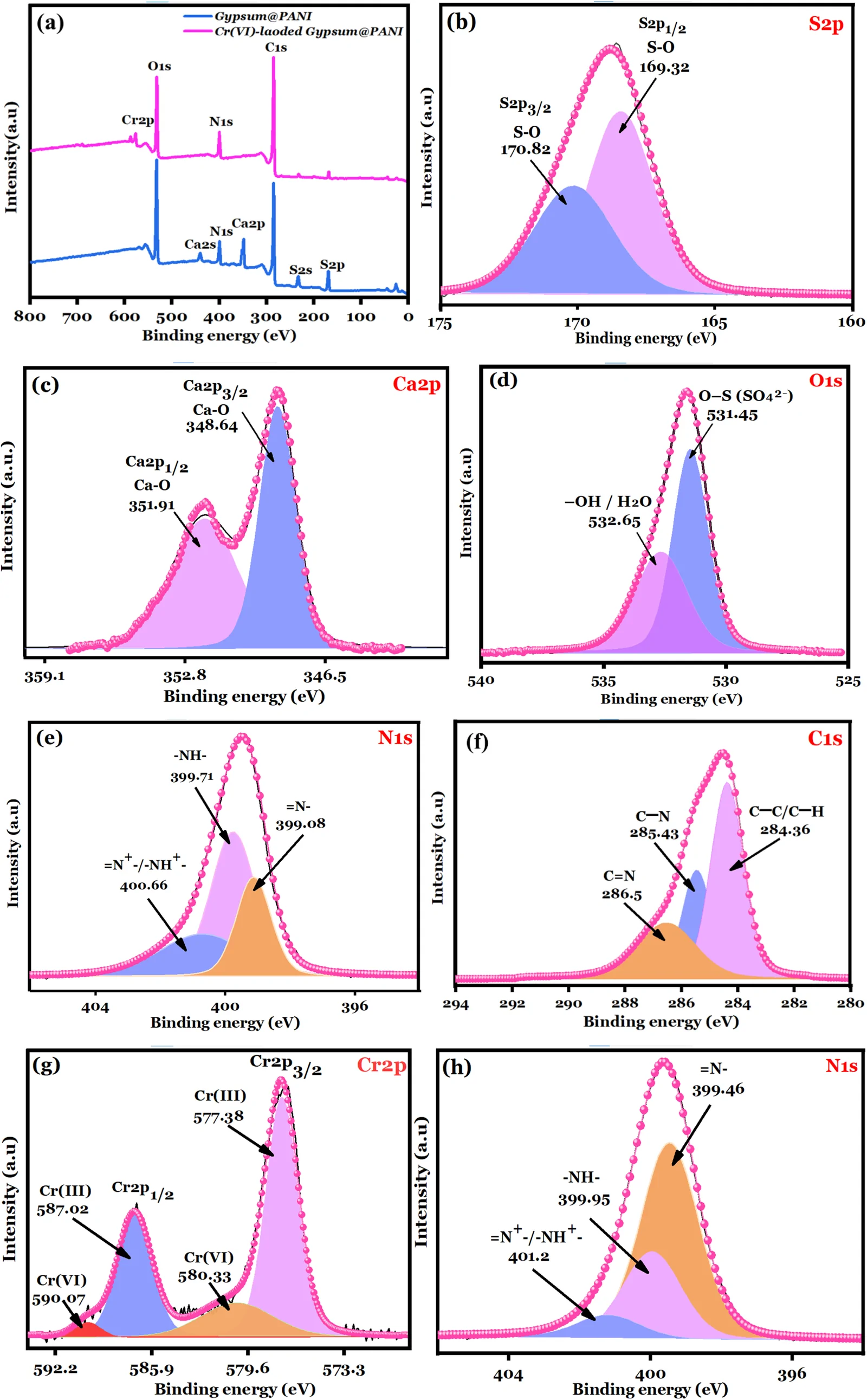
(a) XPS survey spectra of Gypsum@PANI before and after Cr(vi) adsorption; high-resolution spectra of (b) S 2p, (c) Ca 2p, (d) O 1s, and (f) C 1s before adsorption; (e) and (h) N 1s spectra before and after adsorption, respectively; and (g) Cr 2p spectrum of Cr(vi)-loaded Gypsum@PANI.

To unravel the Cr(vi) adsorption–reduction mechanism, XPS characterization of the Gypsum@PANI surface pre- and post-Cr(vi) adsorption is carried out (Fig. 8). XPS full scan of Gypsum@PANI exhibits two intense peaks at 285 and 531 eV, corresponding to C 1s and O 1s, respectively. Other characteristic peaks of S 2p, S 2s, Ca 2p, N 1s, and Ca 2s are observed at 170, 234, 348, 399 and 440 eV, respectively. All these peaks originate from the Gypsum and PANI (Fig. 8(a)).^68^ In the core-level S 2p spectrum (Fig. 8(b)), two peaks of S 2p_3/2_ and S 2p_1/2_ for the S–O bonds in gypsum appear at 169.32 and 170.82 eV, respectively.^69^ From the core-level Ca 2p spectrum (Fig. 8(c)), the Ca 2p_3/2_ and Ca 2p_1/2_ peaks associated with Ca–O in gypsum are detected around 348.64 and 351.91 eV, respectively. The core-level O 1s spectrum (Fig. 8(d)) exhibits two signals at 531.45 and 532.65 eV, which are characteristic of the O–S bonds and adsorbed water, respectively.^70^ As observed in Fig. 8(e), the deconvolution of the high-resolution N 1s spectrum shows that Gypsum@PANI consists of three distinct components. The bands at 399.08, 399.71, and 400.66 eV are ascribed to quinoid imine (=N–), benzoid group (–NH–), and radical cations (=N^+^/–NH^+^–), respectively.^71^ In the XPS spectrum of C 1s (Fig. 8(f)), the signals at 284.36, 285.43, and 286.5 eV are assigned to C–C/C–H, C–N, and C=N of PANI.^72^ Therefore, XPS data confirm the formation of PANI emeraldine salt in Gypsum@PANI.

After Cr(vi) adsorption onto Gypsum@PANI (Fig. 8(g)), the XPS high-resolution scan of Gypsum@PANI displayed two signals ascribed to Cr 2p_1/2_ and Cr 2p_3/2_. These signals were deconvoluted into four components at 577.38 and 587.02 eV for Cr(iii) and at 580.33 and 590.07 eV for Cr(vi). Therefore, the Cr(vi) decontamination involves the simultaneous adsorption and reduction of Cr(vi) to Cr(iii). Besides, an increment in the signal intensity of the =N– band was observed in the N 1s spectrum of Cr(vi)-loaded Gypsum@PANI (Fig. 8(h)). Meanwhile, the intensity of the –NH– peak decreased, indicating oxidation of the PANI emeraldine salt to the pernigraniline form. This further confirms the redox reaction between Cr(vi) and PANI (eqn (20)). The amine groups of PANI serve as electron donors, playing the predominant role in the reduction of Cr(vi) to Cr(iii). Moreover, the peaks of =N– and =N^+^/–NH^+^– groups shift slightly toward higher values. This shift can be ascribed to electrostatic attractions between Cr(vi) and =N^+^/–NH^+^– and the Cr(iii) complexation with =N– (eqn (21)).

20 2HCrO_4_^−^ + 14H^+^ + 3PANi → 2Cr^3+^ + 3PANi^2+^ + 8H_2_O

21 =N– + Cr^3+^ → =N–Cr^3+^

**Fig. 9:**
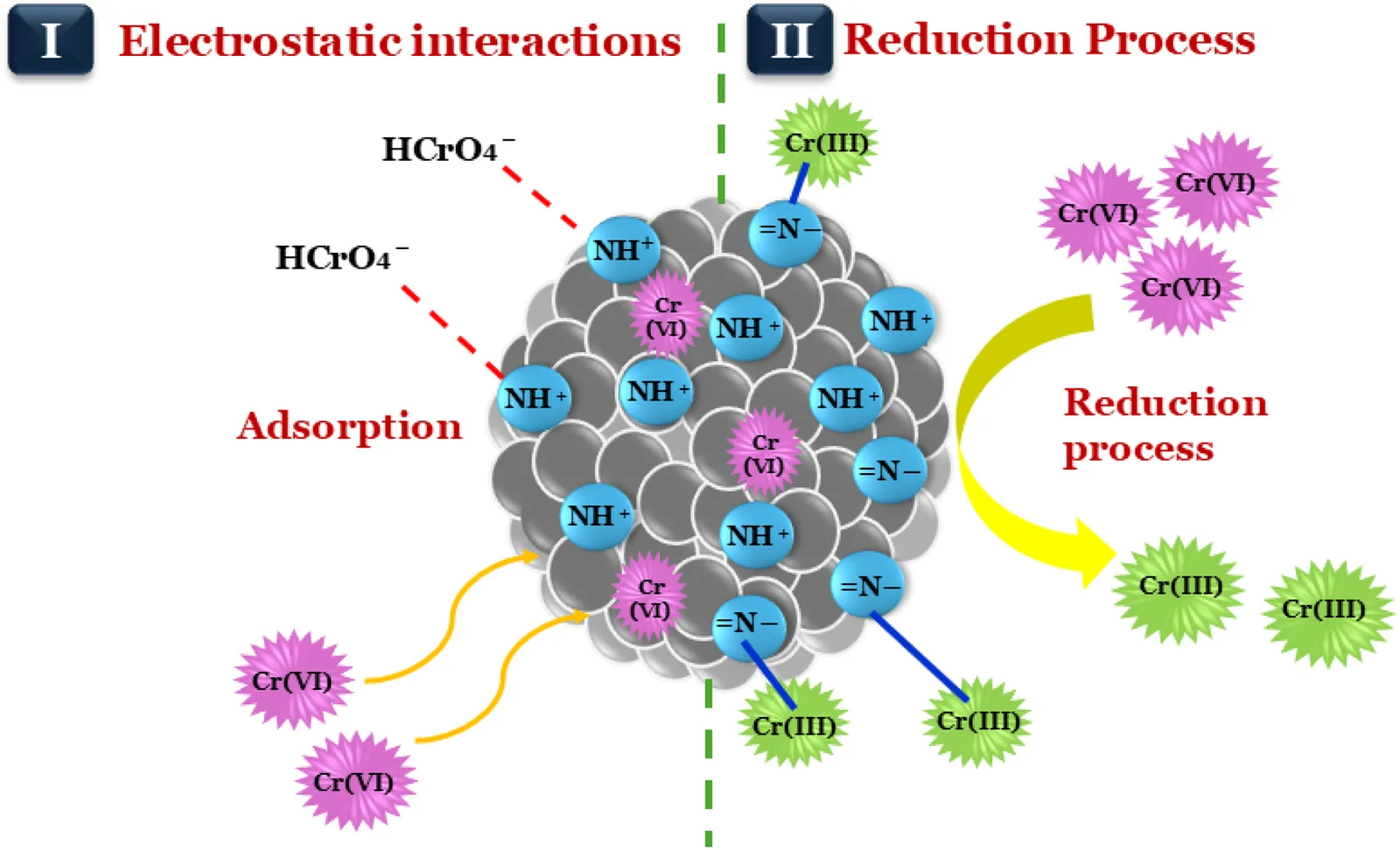
Proposed Cr(vi) detoxification mechanism using Gypsum@PANI.

XPS analysis suggests a two-stage mechanism for Cr(vi) detoxification by Gypsum@PANI. First, Cr(vi) ions are adsorbed on the protonated amine/imine groups of Gypsum@PANI *via* electrostatic attractions. This is followed by the reduction of Cr(vi) to Cr(iii), which is ultimately complexed with the lone pairs of nitrogen atoms.^73^ The Cr(vi) adsorption–reduction mechanism is schematized in Fig. 9.

#### 3.2.8 Impact of interfering anions and Gypsum@PANI reuse

**Fig. 10:**
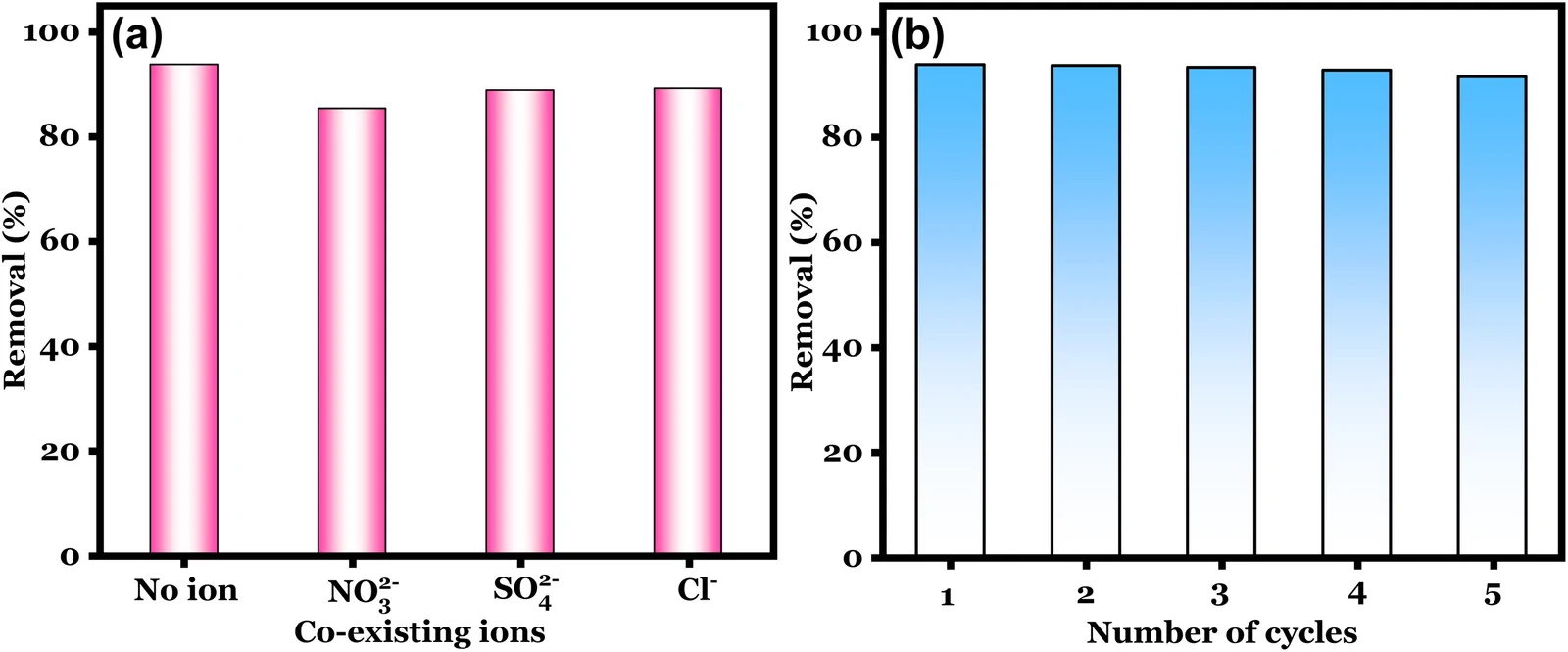
(a) Influence of competing ions on Cr(vi) removal and (b) recycling performance of Gypsum@PANI up to five times (pH 2; equilibrium time 4 h; Gypsum@PANI dosage 0.37 g L^−1^; 20 mg L^−1^ Cr(vi); T 25 °C).

The influence of interfering ions (*e.g.*, Cl^−^, NO_3_^−^ and SO_4_^2−^) on the Cr(vi) removal by Gypsum@PANI was assessed to simulate real wastewater. As shown in Fig. 10(a), the influence of the tested interfering anions is insignificant, except NO_3_^−^. The presence of NO_3_^−^ ions exerted the most pronounced interference, decreasing Cr(vi) elimination yield by approximately 8%. The stronger inhibitory effect of NO_3_^−^ compared with Cl^−^ and SO_4_^2−^ can be attributed to the different reaction mechanisms involved. To better understand the effect of each interfering anion, the thermodynamic feasibility of their reduction reactions was considered. The NO_3_^−^ and SO_4_^2−^ ions can be reduced under acidic conditions (eqn (22)–(24)).^74,75^ Based on the Δ*G*° values of the reduction half-reactions, the Cr(vi) reduction is thermodynamically the most favorable (Δ*G*° = −56.25 kJ mol^−1^), followed by the reduction of NO_3_^−^ to NO_2_^−^ (Δ*G*° = −11.57 kJ mol^−1^) and NO_3_^−^ to NH_4_^+^ (Δ*G*° = −10.13 kJ mol^−1^). However, the reduction of SO_4_^2−^ to HS^−^ is highly unfavorable (Δ*G*° = +385.17 kJ mol^−1^).^74^ These results indicate that SO_4_^2−^ reduction is not expected to occur under experimental conditions. However, the NO_3_^−^ ions can compete with Cr(vi) for available electrons owing to their relatively favorable reduction potential. Consequently, NO_3_^−^ may partially consume the reducing equivalents generated on the Gypsum@PANI surface. This competitive behavior decreases the reduction/adsorption efficiency of Cr(vi). In contrast, Cl^−^ differs fundamentally from NO_3_^−^ and SO_4_^2−^ because it is already in the lowest oxidation state of chlorine (−1). Therefore, Cl^−^ cannot undergo further reduction in aqueous solution.^75^ As a result, Cl^−^ does not compete with Cr(vi) through a redox pathway; rather, its influence is limited to weak electrostatic competition for binding sites. This explains why NO_3_^−^ exhibited a more remarkable inhibitory potency against Cr(vi) removal in comparison with Cl^−^ and SO_4_^2−^. Overall, the Cr(vi) removal performance of Gypsum@PANI remained above 85% in the presence of all tested interfering anions. This result reveals that the Gypsum@PANI composite possesses a greater affinity for Cr(vi), which is beneficial for removing this highly toxic pollutant from contaminated effluents.

22 NO_3_^−^ + 10H^+^ + 8e^−^ → NH_4_^+^ + 3H_2_O (*E*° = +0.882 V)

23 NO_3_^−^ + 2H^+^ + 2e^−^ → NO_2_^−^ + H_2_O (*E*° = +0.837 V)

24 SO_4_^2−^ + 9H^+^ + 8e^−^ → SH^−^ + 4H_2_O (*E*° = +0.248 V)

The safe disposal of spent adsorbents is a critical environmental challenge and requires appropriate remediation strategies. To this end, the reusability of Gypsum@PANI was evaluated over five regeneration cycles. Since Cr(vi) adsorption is most effective under acidic conditions, desorption is favorable at alkaline pH. The regeneration of Gypsum@PANI was performed using a 0.1 M NaOH solution (eluent). As displayed in Fig. 10(b), Gypsum@PANI retains high effectiveness after five reuse times. Only a negligible decrease in performance (from 93% to 91%) was observed during the last cycle. Overall, Gypsum@PANI can be reused repeatedly with only a small loss in Cr(vi) removal performance. To assess the structural stability of Gypsum@PANI after regeneration, the fresh and recycled Gypsum@PANI composites were analyzed by XRD (Fig. S3). As a result, the similarity of the XRD patterns revealed that the Gypsum@PANI composite preserved its structure after reuse. The XRD analysis demonstrates the good structural stability of the Gypsum@PANI composite.

## 4 Conclusion

In summary, a novel mesoporous Gypsum@PANI composite was successfully fabricated for Cr(vi) detoxification. The prominent Cr(vi) adsorption efficiency was achieved at pH 2, aligning with previous studies in the field. The experimental results were satisfactorily aligned with the Freundlich and PSO equations. The Gypsum@PANI composite exhibits an impressive Cr(vi) uptake capacity of 310.36 mg g^−1^. According to the RSM-BBD study, the highest Cr(vi) removal yield of 93.86% was achieved at pH 2 using a Gypsum@PANI dosage of 0.35 g L^−1^ and Cr(vi) concentration of 20 mg L^−1^ for 4 h at 25 °C. The ASP2 model was adopted to predict Cr(vi) adsorption. Stereographic analysis indicated that the Cr(vi) adsorption proceeds *via* multi-anchorage and multi-molecular mechanisms. Moreover, the thermodynamic functions proved that Cr(vi) adsorption was a feasible and energy-releasing process characterized by an increment in disorder. The mechanism of Cr(vi) detoxification can be explained by adsorption *via* electrostatic attractions, followed by Cr(vi) reduction into Cr(iii). This detoxification process enhances the environmental safety of the treated water. Regeneration tests demonstrated that Gypsum@PANI preserved its high effectiveness after five reuses. Overall, the as-developed Gypsum@PANI composite undoubtedly contributes to the progress in developing practical solutions for the remediation of Cr(vi)-laden wastewater.

## Conflicts of interest

The authors declare that they have no competing financial interests or personal relationships that could have influenced the work reported in this paper.

## Supplementary Material

RA-016-D6RA04236H-s001

## Data Availability

All data underlying the results are available as part of the article, and no additional source data are required. Supplementary information (SI) is available. See DOI: https://doi.org/10.1039/d6ra04236h.
