# Supplementary material for: Adsorption-synergized reduction of Cr(vi) using Gypsum@PANI composite: unraveling mechanism and advanced statistical physics prediction
Source: RSC Adv. 2026 Aug 4;16(39):44567–84. doi: 10.1039/d6ra04236h (PMC13434751; doi:10.1039/d6ra04236h)
Supplement: Supplementary file 1 [file RA-016-D6RA04236H-s001.pdf]

Supplementary Information (SI) for RSC Advances.
This journal is © The Royal Society of Chemistry 2026

Adsorption-Synergized Reduction of Cr(VI) using Gypsum@PANI Composite: Unraveling Mechanism and Advanced Statistical Physics Prediction

Samira El Omari[a,b,*], Kamal Ait El Bacha[a], Abdelaziz Imgharn[a], Oscar May Tzuc[c], Youness Abdellaoui[d,e], Abdallah Albourine[a,f], Karim Benhabib[b], Mohamed Laabd[a,*]

[a] Laboratory of Materials and Environment, Faculty of Sciences, Ibnou Zohr University, Agadir, Morocco.

[b] Eco-Process, Optimization and Decision Support. Jules Verne University of Picardie (EPROAD, UR), France.

[c] Faculty of Engineering, Autonomous University of Campeche, Campus V, Av Humberto Lanz, Col. Ex Hacienda Kalá, C.P. 24085, San Francisco de Campeche, Campeche, Mexico.

[d] Cinvestav Saltillo. Sustainability of Natural Resources and Energy, Av. Industria Metalúrgica 1062, Parque Industrial Ramos Arizpe. Ramos Arizpe, Coahuila C.P. 25900, Mexico.

[e] CONAHCyT-Cinvestav Saltillo. Sustainability of Natural Resources and Energy, Av. Industria Metalúrgica 1062, Parque Industrial Ramos Arizpe. Ramos Arizpe, Coahuila C.P. 25900, Mexico.

[f] Laboratory of Industrial Engineering, Energy and Environment (LI3E), SupMTI Rabat, Rabat 10000, Morocco.

[*]Corresponding authors. samiraelomari.00@gmail.com (S. El Omari) & m.laabd@uiz.ac.ma (M. Laabd)

Supporting information

Figures

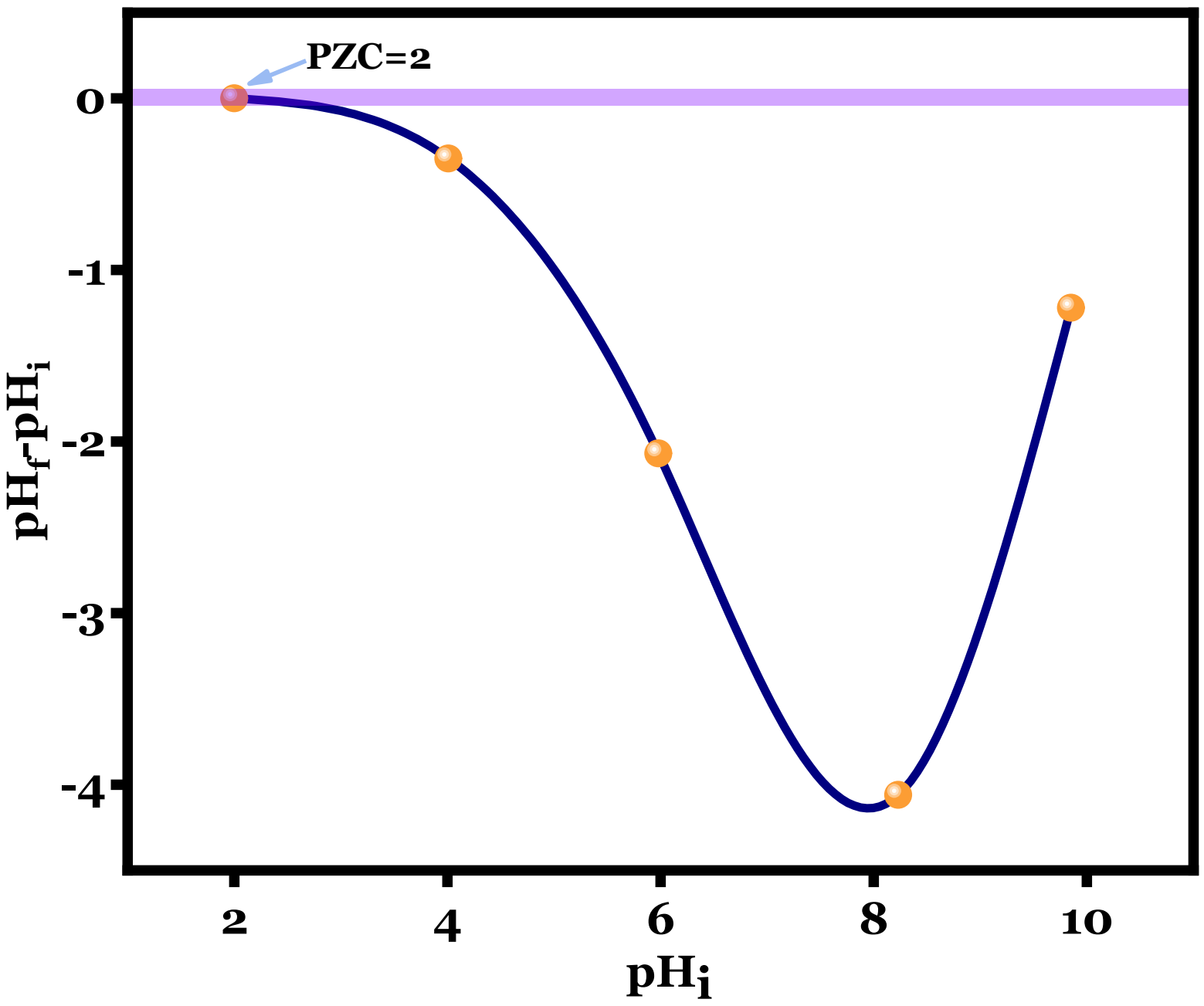

Fig. S1. pH_{PNC} determination of Gypsum@PANI.

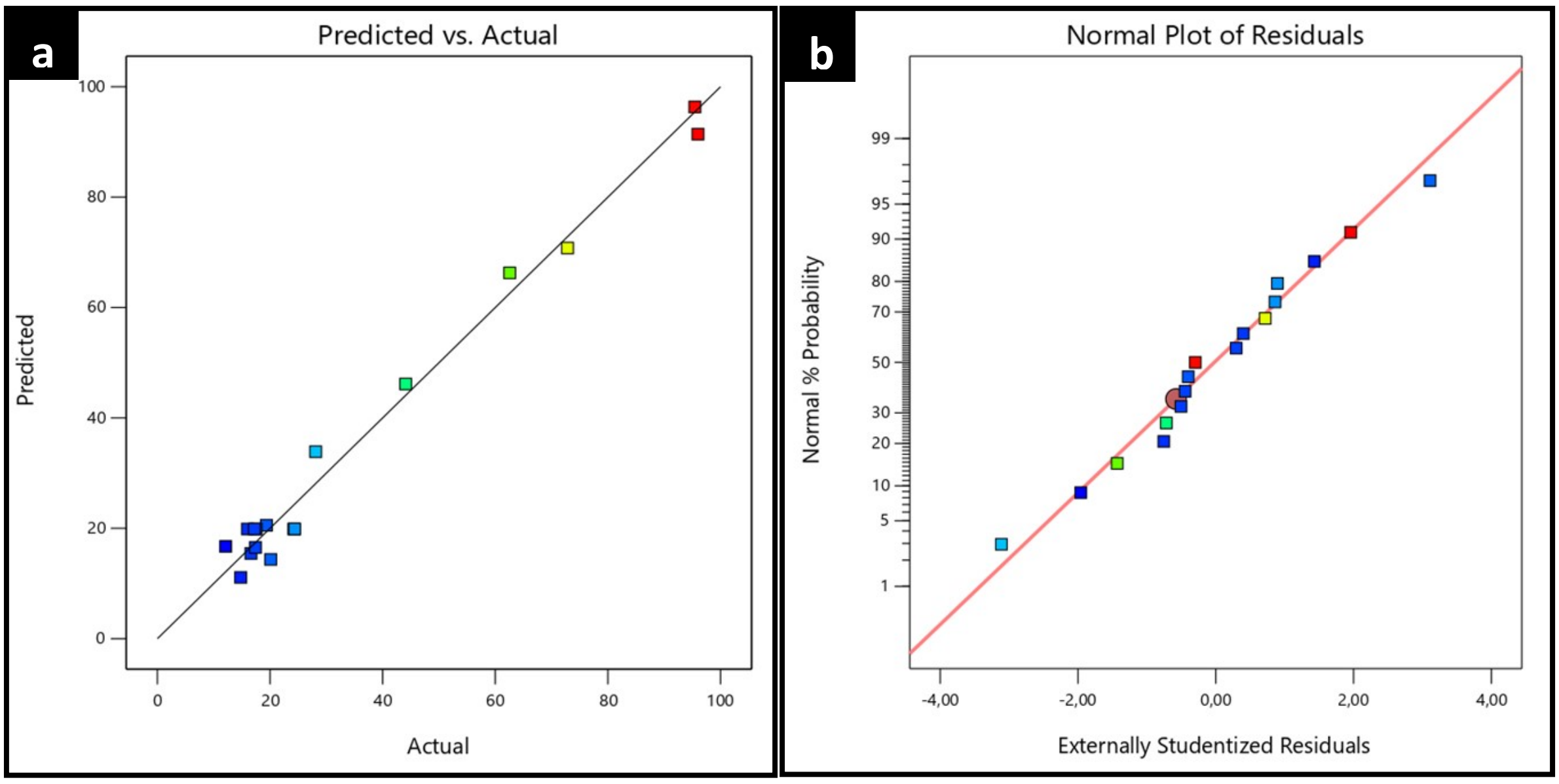

Fig. S2. **(a)** Observed vs. predicted % removal and **(b)** normal probability vs. studentized residuals for Cr(VI) adsorption onto Gypsum@PANI.

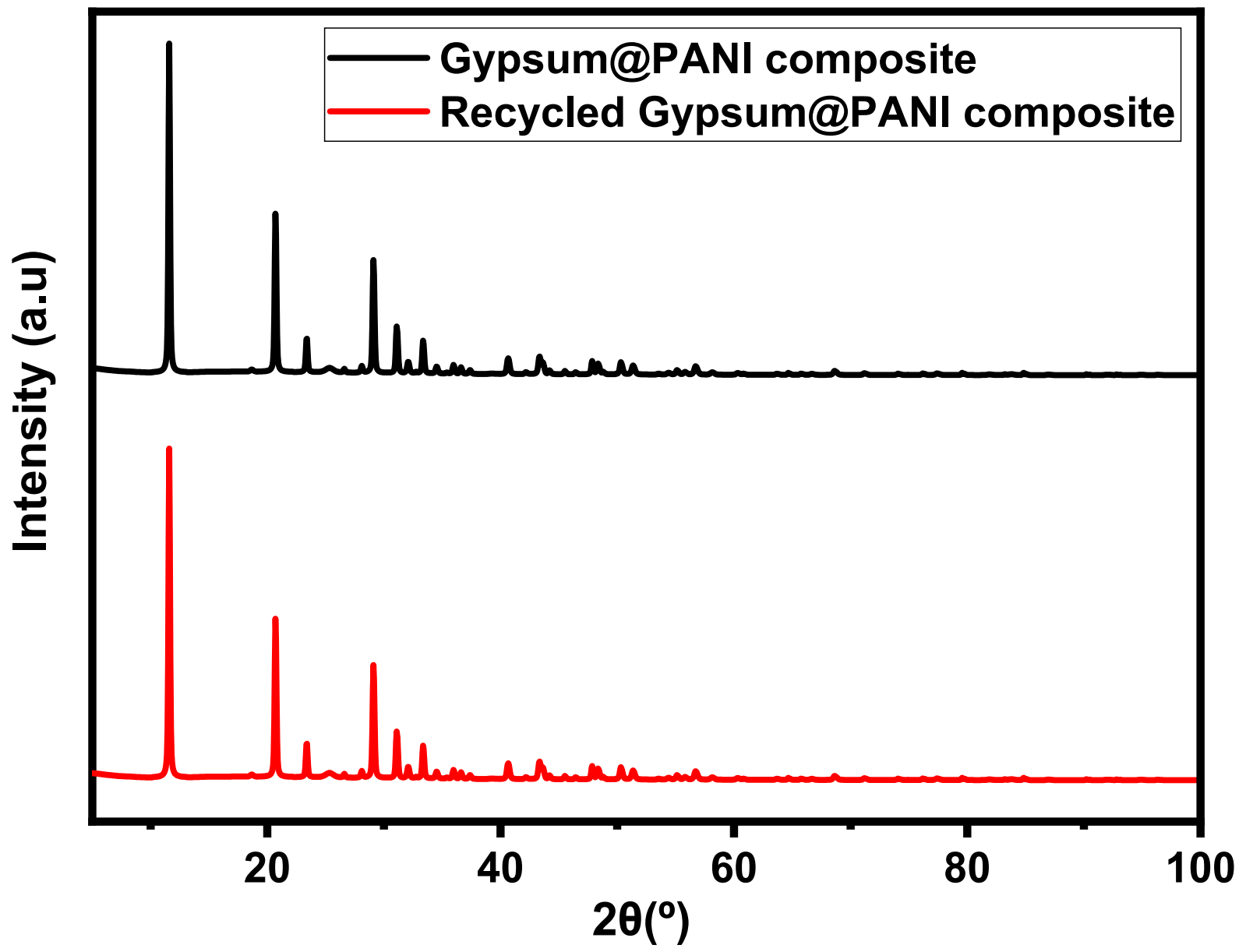

Fig S3. XRD diffractograms of the Gypsum@PANI composite before and after regeneration.

Tables

Table S1. BBD-RSM design and actual results for Cr(VI) adsorption onto Gypsum@PANI.

Run	Operational conditions			Response
	Gypsum@PANI dose (g/L)	pH	Cr(VI) concentration (mg/L)	R (%)
1	-1	-1	0	16.63
2	1	-1	0	62.57
3	-1	1	0	14.8
4	1	1	0	19.37
5	-1	0	-1	28.12
6	1	0	-1	95.45
7	-1	0	1	17.41
8	1	0	1	20.13
9	0	-1	-1	96.01
10	0	1	-1	72.84
11	0	-1	1	44.09
12	0	1	1	12.14
13	0	0	0	24.24
14	0	0	0	24.39
15	0	0	0	16.02
16	0	0	0	17.54
17	0	0	0	17.24
